# Malaria transmission and prevalence in rice-growing versus non-rice-growing villages in Africa: a systematic review and meta-analysis

**DOI:** 10.1016/S2542-5196(21)00349-1

**Published:** 2022-03-09

**Authors:** Kallista Chan, Lucy S Tusting, Christian Bottomley, Kazuki Saito, Rousseau Djouaka, Jo Lines

**Affiliations:** a Department of Disease Control, London School of Hygiene & Tropical Medicine, London, UK; b Centre on Climate Change and Planetary Health, London School of Hygiene & Tropical Medicine, London, UK; c Department of Infectious Disease Epidemiology, London School of Hygiene & Tropical Medicine, London, UK; d Africa Rice Center, Bouake, Côte d'Ivoire; e International Institute of Tropical Agriculture, Cotonou, Benin

## Abstract

**Background:**

Rice fields in Africa are major breeding sites for malaria vectors. However, when reviewed in the 1990s, in settings where transmission was relatively intense, there was no tendency for malaria indices to be higher in villages with irrigated rice fields than in those without. Subsequently, intervention coverage in sub-Saharan Africa has been massively scaled up and malaria infection prevalence has halved. We re-examined this rice–malaria relationship to assess whether, with lower malaria transmission, malaria risk is greater in rice-growing than in non-rice-growing areas.

**Methods:**

For this systematic review and meta-analysis, we searched EMBASE, Global Health, PubMed, Scopus, and Web of Science to identify observational studies published between Jan 1, 1900, and Sept 18, 2020. Studies were considered eligible if they were observational studies (cross-sectional, case-control, or cohort) comparing epidemiological or entomological outcomes of interest between people living in rice-growing and non-rice-growing rural communities in sub-Saharan Africa. Studies with pregnant women, displaced people, and military personnel as participants were excluded because they were considered not representative of a typical community. Data were extracted with use of a standardised data extraction form. The primary outcomes were parasite prevalence (*P falciparum* parasite rate age-standardised to 2–10-year-olds, calculated from total numbers of participants and number of infections [confirmed by microscopy or rapid diagnostic test] in each group) and clinical malaria incidence (number of diagnoses [fever with *Plasmodium* parasitaemia confirmed by microscopy or rapid diagnostic test] per 1000 person-days in each group). We did random-effects meta-analyses to estimate the pooled risk ratio (RR) for malaria parasite prevalence and incidence rate ratio (IRR) for clinical malaria in rice-growing versus non-rice-growing villages. RRs were compared in studies conducted before and after 2003 (chosen to mark the start of the mass scale-up of antimalaria interventions). This study is registered with PROSPERO (CRD42020204936).

**Findings:**

Of the 2913 unique studies identified and screened, 53 studies (including 113 160 participants across 14 African countries) were eligible for inclusion. In studies done before 2003, malaria parasite prevalence was not significantly different in rice-growing versus non-rice-growing villages (pooled RR 0·82 [95% CI 0·63–1·06]; 16 studies, 99 574 participants); however, in post-2003 studies, prevalence was significantly higher in rice-growing versus non-rice growing villages (1·73 [1·01–2·96]; seven studies, 14 002 participants). Clinical malaria incidence was not associated with residence in rice-growing versus non-rice-growing areas (IRR 0·75 [95% CI 0·47–1·18], four studies, 77 890). Potential limitations of this study include its basis on observational studies (with evidence quality rated as very low according to the GRADE approach), as well as its omission for the effects of seasonality and type of rice being cultivated. Risk of bias and inconsistencies was relatively serious, with *I*^2^ greater than 90% indicating considerable heterogeneity.

**Interpretation:**

Irrigated rice-growing communities in sub-Saharan Africa are exposed to greater malaria risk, as well as more mosquitoes. As increasing rice production and eliminating malaria are two major development goals in Africa, there is an urgent need to improve methods for growing rice without producing mosquitoes.

**Funding:**

Wellcome Trust Our Planet Our Health programme, CGIAR Agriculture for Nutrition and Health.

## Introduction

Rice cultivation and malaria are linked in sub-Saharan Africa because of two biological characteristics of the most important African mosquito vector, *Anopheles gambiae* sensu lato (sl; the species complex referring to *Anopheles gambiae* sensu stricto [ss], *Anopheles coluzzii,* and *Anopheles arabiensis*). The first of these characteristics is that adults of this species complex are long-lived and prefer to bite humans, making them exceptionally efficient in transmitting malaria;^1^ this fact is why Africa accounts for 96% of the world's malaria mortality burden, with approximately 602 000 of the 627 000 global malaria deaths occurring in the region in 2020.^2^ The second characteristic is that the larvae of *A gambiae* sl are very well adapted to, and can breed abundantly in, the aquatic conditions in rice fields.^3^

**Research in context**

**Evidence before this study**

Rice fields in Africa are major breeding sites for malaria vectors, bringing greater abundance of *Anopheles* species in rice-growing villages. When reviewed two decades ago, it was observed that these extra mosquito vectors did not increase the incidence of malaria in humans, and some reductions in malaria infection prevalence were observed. Since then, antimalarial intervention coverage across sub-Saharan Africa has greatly increased and become more equitable, and malaria infection prevalence has halved, calling for a re-examination of this rice–malaria relationship. Between May, 23, 2018, and Sept 18, 2020, we searched EMBASE, Global Health, PubMed, Scopus, and Web of Science, without restrictions on language or date of publication, to identify community-based studies that compared malaria risk between rice-growing and non-rice-growing areas in sub-Saharan Africa. Combinations of the following keywords were used: malaria, *Plasmodium falciparum*, *Anopheles*, mosquito, prevalence, incidence, risk, Africa, rice, paddies, paddy, irrigation, human biting rate, sporozoite rate, and entomological inoculation rate. Risk of bias of eligible studies was generally of an intermediate level.

**Added value of this study**

In this systematic review and meta-analysis, by comparing older studies included in previous reviews with more recently published studies, we assessed whether the decline in malaria transmission has changed the associations between rice cultivation and malaria risk. It was confirmed that before the year 2003, infection prevalence was not higher in rice-growing communities. However, after 2003, malaria prevalence was almost two times higher in rice-growing communities. It was also confirmed that as underlying malaria intensity decreased, there was an increase in the strength of the association between rice cultivation and malaria risk. Malaria transmission (measured as the rate of infective biting on exposed residents) was also greater in rice-growing areas, indicating that although rice-field malaria vectors might have somewhat lower sporozoite rates, this reduction does not compensate for their substantially greater numbers.

**Implications of all the available evidence**

African ministries of health are considering how to eliminate malaria, while ministries of agriculture are actively planning the expansion and intensification of irrigated rice production. These objectives are both desirable, but our updated review indicates that the latter process might interfere with the former, as rice cultivation brings increased malaria risk. To reconcile these two goals, African countries urgently need to develop and promote methods of growing rice without growing malaria vectors.

Against this biological background, in many African countries, ministries of agriculture and their partners are planning for a massive expansion in irrigated rice cultivation, in response to rapidly increasing consumer demand.^4^ Rice is the fastest growing food in Africa; harvested areas increased by over 600% from 1961 to 2019.^5^ Meanwhile, ministries of health and their partners are working towards the eventual elimination of malaria. Therefore, it is important to consider the potential interactions between these two development processes, and whether they might interfere with one another.

The links between rice and malaria were studied in a series of case studies in west and east Africa during the 1990s and early 2000s.^6^, ^7^ An overall review of the findings revealed that, although mosquito vectors (especially *A gambiae* sl) were substantially more abundant in villages beside irrigated rice fields than in nearby non-rice-growing areas, the prevalence of malaria in rice-growing villages was unexpectedly either the same as or slightly lower than that in non-rice-growing control communities. Ijumba and Lindsay coined the term “paddies paradox” to describe this phenomenon.^6^ Investigations into the possible causes of this paradox suggested that, in many cases, rice cultivation also brought substantial economic benefits, particularly improvements to family income (and hence better access to commercial mosquito nets and antimalarial drugs) and community infrastructure (housing, transport, and health services). Thus, families could protect themselves and respond to malaria episodes more promptly and effectively.^8^ Density-dependent effects could also contribute: some studies^9^, ^10^, ^11^, ^12^, ^13^ found a reduction in vectorial capacity at high mosquito densities through reduced adult longevity (probably due to greater larval competition) and reduced blood feeding success (probably due to greater use of bed nets). For 20 years, this paradoxical conclusion has helped to reassure rice experts in Africa that they are contributing to development and not making the malaria problem worse.^14^

We were prompted to re-examine this conclusion because the malaria situation across sub-Saharan Africa has changed radically in the past two decades. The massive upscaling in coverage of modern malaria control interventions (such as insecticide-treated nets and antimalarial drugs) has greatly reduced the intensity of transmission for most of the at-risk population in Africa, where the population exposed to hyperendemic or holoendemic transmission has fallen from 33% to 9%.^15^ Moreover, there is clear evidence that intervention scale-up has reduced previous inequities in bed-net coverage, suggesting less severe inequality between rice-growing and non-rice-growing villages.^16^ Furthermore, the paddies paradox was often interpreted as an implication that the extra mosquitoes from rice fields were generally harmless, which was misleading. Therefore, we re-examined whether these recent changes in malaria epidemiology have altered the relationship between malaria risk and irrigated-rice cultivation in Africa.

## Methods

### Search strategy and selection criteria

We did a systematic review and meta-analysis following the PRISMA reporting guidelines.^17^ EMBASE, Global Health, PubMed, Scopus, and Web of Science were searched without language restrictions to identify studies published between Jan 1, 1900, and Sept 18, 2020 (the end date of our search). Combinations of the following keywords and Medical Subject Headings were used: malaria, *Plasmodium falciparum*, prevalence, incidence, risk, Africa, rice, padd*, and irrigation. The full search strategy is summarised in the appendix (p 1). Additional references were identified using citation searches of obtained articles, conference proceedings (such as the Multilateral Initiative on Malaria's Pan-African Malaria Conferences and the American Society of Tropical Medicine and Hygiene), and contact with authors.

The inclusion criteria for epidemiological studies were as follows: studies with participants of any age residing in sub-Saharan Africa; studies with a cross-sectional, case-control, or cohort design; studies conducted in rice-growing and non-rice-growing areas; and studies reporting on any epidemiological outcomes of interest (parasite prevalence or malaria incidence). Studies with pregnant women, displaced people, and military personnel as participants were excluded because they were considered not representative of a typical community. The inclusion criteria for entomological studies were as follows: studies with a cross-sectional, case-control, or cohort design; studies conducted in rice-growing and non-rice-growing areas; and studies reporting on any entomological outcomes of interest (human biting rate, sporozoite rate, and entomological inoculation rate), reported as summary estimates. The titles and abstracts of studies identified by the searches were screened by KC and JL, and, for those that were potentially relevant, full texts were assessed. Any conflicts were resolved by LT.

The protocol for this study is available online.

### Data analysis

Data on the following study variables were extracted using a predefined and standardised form: participants (age and recruitment methods), sampling method (ie, type of mosquito trap and ascertainment of malaria positivity [microscopy or rapid diagnostic test]), exposures (ie, residence in rice-growing or non-rice-growing area), comparisons (type of rice growing [number of cropping seasons] *vs* type of non-rice-growing area [control area]), epidemiological and entomological outcomes (parasite prevalence, malaria incidence, human biting rate, sporozoite rate, entomological inoculation rate), summary measures (odds ratio [OR], risk ratio [RR], and incidence rate ratio [IRR], including adjusted values), study design, setting (physical environment [ie, semi-arid, forest, highlands, coastal]), sample size, vector species, long-lasting insecticidal net and indoor residual spraying coverage, and malaria transmission intensity. Data were extracted by KC and a 10% sub-sample was randomly selected for validation by JL. Any duplicate data (ie, multiple reports from the same study) were excluded.

The primary outcomes were epidemiological outcomes in human participants: parasite prevalence (confirmed by microscopy or rapid diagnostic test, in any age group) and malaria incidence (fever with *Plasmodium* parasitaemia confirmed by microscopy or rapid diagnostic test, in any age group). Secondary outcomes were entomological indices of interest: human biting rate (the number of mosquitoes in contact with a person per night), sporozoite rate (the percentage of female *Anopheles* mosquitoes with sporozoites in the salivary glands), and entomological inoculation rate (the estimated number of infective bites per person per year, which is a product of human biting rate and sporozoite rate). Indoor and outdoor human landing catches were considered the gold standard for measuring entomological outcomes, followed by Centers for Disease Control and Prevention (CDC) light traps or pyrethrum spray catches.

For continuous outcomes (human biting rate and entomological inoculation rate), the arithmetic or geometric means, corresponding SDs or SEs, and number of participants in exposed and control groups were extracted. For dichotomous outcomes (sporozoite rate and parasite prevalence), the total numbers of participants and events in each group were extracted. For count data (clinical malaria episodes), the number of events and the total person-time at risk in each group were extracted. Adjusted effect sizes of entomological and epidemiological outcomes, where reported, were also extracted. Study authors were contacted for missing data.

Analyses were structured first by outcome, second by vector species (if applicable), and third by study design. All eligible studies were included in a qualitative analysis. Studies were also analysed semiquantitatively if sufficient data to calculate crude effects were reported (but 95% CIs were not reported) and quantitatively if crude or adjusted effects with 95% CIs were reported. Because age is an important source of heterogeneity in parasite prevalence data, *P falciparum* parasite rates were age-standardised to 2–10-year-olds (*Pf*PR_2–10_) to enable study comparability using a modified Pull and Grab algorithm, via an R package called ageStand.^18^

Entomological and epidemiological data were combined in meta-analyses via the R metafor package.^19^ Regardless of heterogeneity (*I*^2^), random-effects models were used to calculate pooled (crude or adjusted) effect measures from quantitative studies only (ratio of means [ROM] for quantitative outcomes, RR for dichotomous outcomes, and IRR for clinical malaria), as well as corresponding 95% CIs, to illustrate the effect of rice cultivation on each outcome of each study. Separate meta-analyses were done for crude and adjusted results.

To evaluate the effect of the recent changes in malaria on the rice–malaria relationship, effect sizes were analysed in two ways. First, we did a subgroup analysis in which studies were separated by whether they were done before 2003 or from 2003 onwards; this cut-off year was chosen partly because it was the time at which previous reviews reached the paddies paradox conclusion, but mainly because it was when intervention scale-up started.^20^ Antimalarial interventions started scaling up in sub-Saharan Africa between 2001 and 2005, varying between countries, and so 2003 was chosen as the midpoint to represent this change. A sensitivity analysis between these years (2001 and 2005) was done to evaluate the robustness of the year 2003 as a cut-off point. Second, a Pearson's correlation test was done between study effect sizes (log-transformed) and their underlying malaria intensity (parasite prevalence in the control group). Results from the meta-analyses and subgroup analyses were depicted using bar graphs.

Risk of bias for cross-sectional and cohort studies was assessed using the Newcastle-Ottawa Scale.^21^ Publication bias was assessed by the visual inspection of funnel plots and the Egger's test for funnel plot asymmetry.^22^ Quality and strength of the evidence were evaluated using the Grading of Recommendations, Assessment, Development and Evaluation (GRADE) approach.^23^

This study is registered with the International Prospective Register of Systematic Reviews (CRD42020204936).

### Role of the funding source

The funder of the study had no role in study design, data collection, data analysis, data interpretation, or writing of the report.

## Results

**Figure 1:**
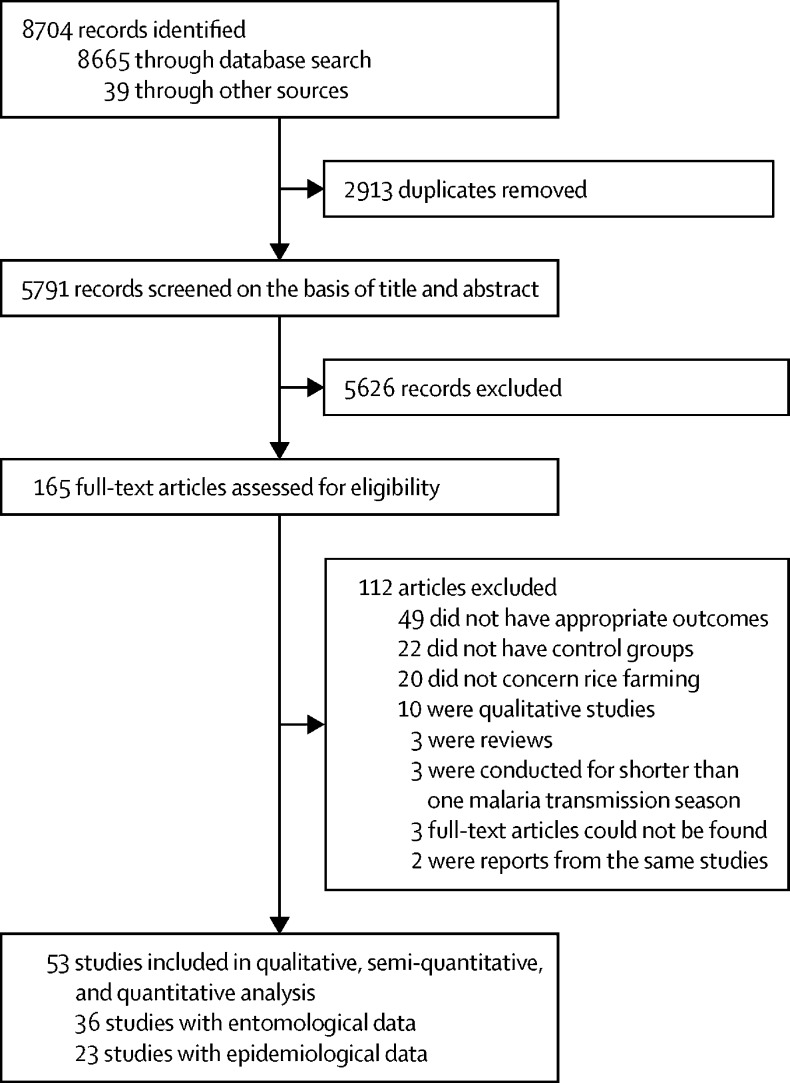
Study

Our search yielded 2913 studies after removal of duplicates (figure 1). 53 studies^8^, ^24^, ^25^, ^26^, ^26^, ^27^, ^28^, ^29^, ^30^, ^31^, ^32^, ^33^, ^34^, ^35^, ^36^, ^37^, ^38^, ^39^, ^40^, ^41^, ^42^, ^43^, ^44^, ^45^, ^46^, ^47^, ^48^, ^49^, ^50^, ^51^, ^52^, ^53^, ^54^, ^55^, ^56^, ^57^, ^58^, ^59^, ^60^, ^61^, ^62^, ^63^, ^64^, ^65^, ^66^, ^67^, ^68^, ^69^, ^70^, ^71^, ^72^, ^73^, ^74^, ^75^ (with a total of 113 160 participants) met the inclusion criteria, various subsets of which were included in the quantitative, semi-quantitative, and qualitative analyses depending on the outcome of interest (appendix pp 2–7). 23 (43%) studies reported data on parasite prevalence, five (9%) on malaria incidence, 36 (68%) on human biting rate, 22 (42%) on sporozoite rate, and 19 (36%) on entomological inoculation rate. A description of the included studies can be found in the appendix (pp 2–7). All studies were conducted between 1971 and 2016 in rural settings across 14 sub-Saharan African countries. 27 studies were done in west Africa (eight countries), six studies in central Africa (Cameroon), and 20 studies in east Africa (five countries). Descriptions of study areas reported that the type of rice grown varied, and included swamps, rain-fed rice, (small-scale) traditional flooded irrigated rice, and (large-scale) rice irrigation schemes. Control villages were usually 5–20 km away from rice-growing villages and engaged in traditional crop farming, market gardening, sugar plantations, pastoralism, or were savannah areas and inland valleys without rice cultivation.

**Figure 2:**
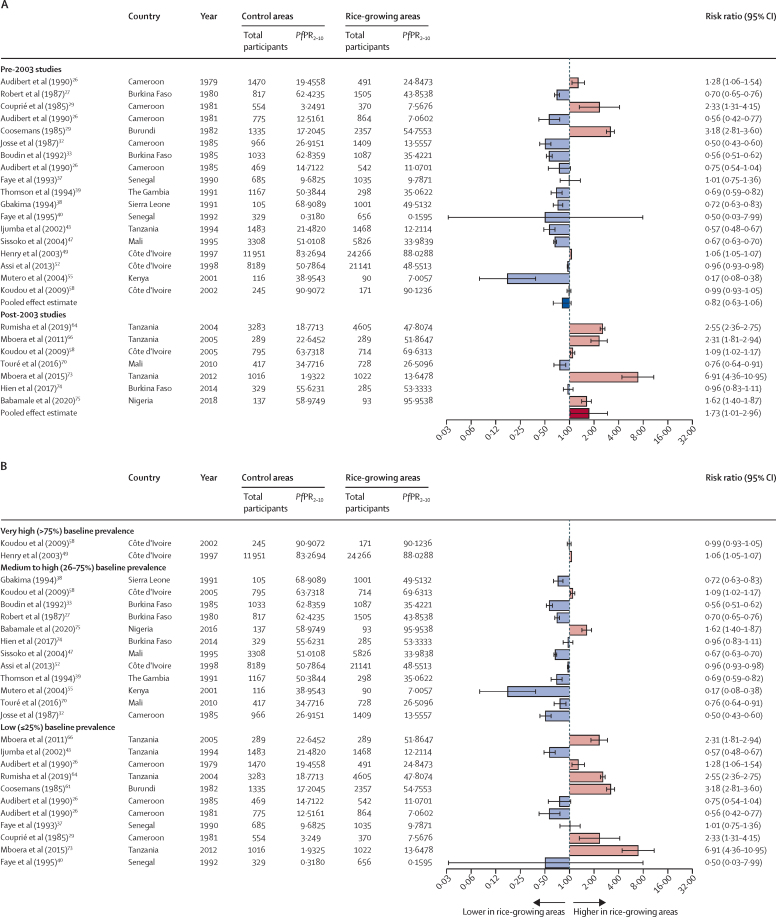

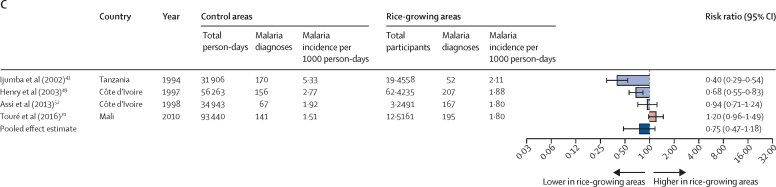
Meta-analyses of the association between residence in rice-growing areas and malaria epidemiological outcomes Crude risk ratios for malaria infection prevalence (*Pf*PR_2–10_) plotted ordered by year of study and subgroup (before and after 2003) (A), and by underlying malaria intensity (*Pf*PR_2–10_ in control group) (B). An increase in effect size was found with decreasing malaria prevalence in the control (non-rice-growing) villages (coefficient –0·417 [95% CI –0·688 to –0·034], p=0·038). (C) Crude incidence rate ratios for clinical malaria incidence (per 1000 person-days) ordered by year of study. Pooled effect estimates based on quantitative studies, calculated using random-effects models, are presented at the bottom of the graphs (and separately for each subgroup in panel A). Error bars are 95% CIs. *Pf*PR_2–10_=*Plasmodium falciparum* parasite rate age-standardised to 2–10-years age group.

22 studies reported malaria prevalence in rice-growing and non-rice-growing villages and were included in the meta-analysis, with 16 studies^26^, ^27^, ^29^, ^30^, ^32^, ^33^, ^37^, ^38^, ^39^, ^40^, ^43^, ^47^, ^49^, ^52^, ^55^, ^58^ conducted before 2003 and seven studies^58^, ^64^, ^66^, ^70^, ^73^, ^74^, ^75^ since 2003 (one study included analyses both before and since 2003; figure 2A). Before 2003, rice-growing was not associated with increased malaria prevalence (crude RR 0·82 [95% CI 0·63–1·06], 16 studies, 99 574 participants; adjusted OR [aOR] 0·73 [95% CI 0·57–0·89], two studies, 11 955 participants; appendix p 8).^49^, ^52^ From 2003 onwards, however, there was a 73% greater risk of malaria infection in rice-growing than in control villages (1·73 [1·01–2·96], seven studies, 14 002 participants; 7·69 [2·72–12·66], one study, 1019 participants).^73^ A Wald-type test indicated that the pooled RR estimated from studies conducted since 2003 was significantly different from that of studies before 2003 (p=0·014). The sensitivity analysis found that 2003 was a robust year to mark the start of the scale-up of interventions; the pooled RRs from post-scale-up studies were unaffected by the choice of the cut-off year, but the pre-scale-up RR moved towards the null as cut-off year increased (appendix p 8).

When we assessed whether the effect of rice growing on malaria was influenced by the underlying malaria intensity (*Pf*PR_2–10_), we found an increase in effect size with decreasing malaria prevalence in the control (non-rice-growing) villages (coefficient –0·417 [95% CI –0·688 to –0·034], p=0·038). Where malaria prevalence was very high (>75%) in control villages, there was almost no difference in prevalence in rice-growing villages; areas where prevalence was medium to high (26–75%) in control villages mostly had a lower prevalence in rice-growing villages; and, conversely, in areas with low prevalence (≤25%) in control villages, malaria risk was usually higher in rice-growing villages (figure 2B).

There was no association between rice cultivation and clinical malaria (IRR 0·75 [95% CI 0·47–1·18], four studies, 77 890 participants; figure 2C).^43^, ^49^, ^52^, ^70^

**Figure 3:**
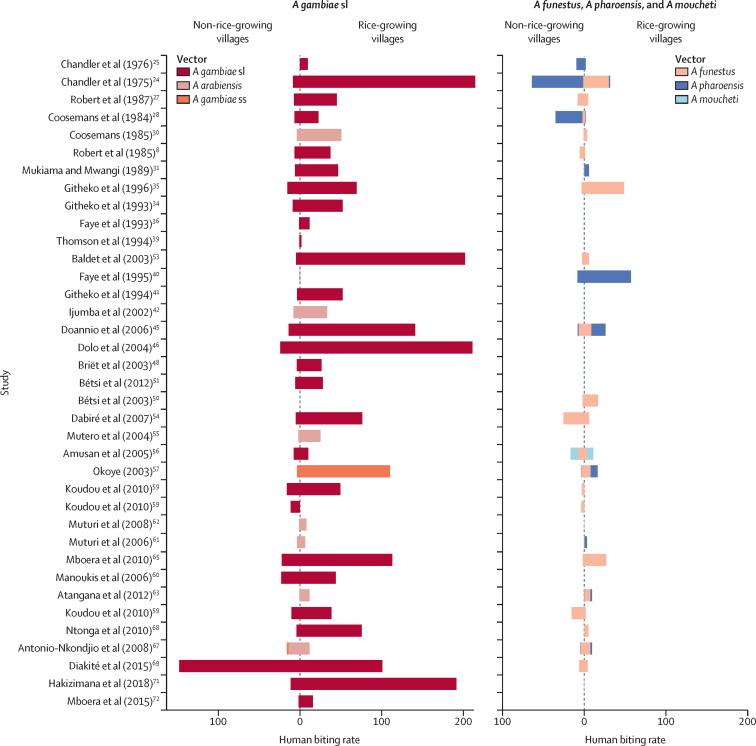
Human biting rate in non-rice-growing and rice-growing villages Comparison of the human biting rate (mosquitoes per person per night) of major malaria vectors in non-rice-growing and rice-growing villages in Africa, by vector species (*Anopheles gambiae* sl [including *Anopheles arabiensis* and *Anopheles gambiae* ss] and other species [*Anopheles funestus*, *Anopheles pharoensis*, and *Anopheles moucheti*]). Studies are ordered by year of study (some studies had data for more than one year). In most instances, *A gambiae* sl was the dominant species in rice-growing areas and *A funestus* and *A pharoensis* were found in lower densities.

36 studies collected entomological outcomes, all of which reported comparative figures on *Anopheles* human biting rates in rice-growing and non-rice-growing villages. Human biting rates were mostly measured directly using human landing catches (27 studies), and, in some circumstances, indirectly using CDC light traps (seven studies) or pyrethrum spray catches (two studies). In most studies (n=35), *A gambiae* sl was the dominant vector, followed by *Anopheles funestus* and *Anopheles pharoensis* (figure 3). It was not determined which sibling species of the *A gambiae* sl species complex was predominant because only eight studies conducted identification to that level. Where sibling species identification was done, the dominant species were *A arabiensis* in Cameroon and east Africa (seven studies), and *A gambiae* ss (molecular form unknown) in Nigeria (one study).

**Figure 4:**
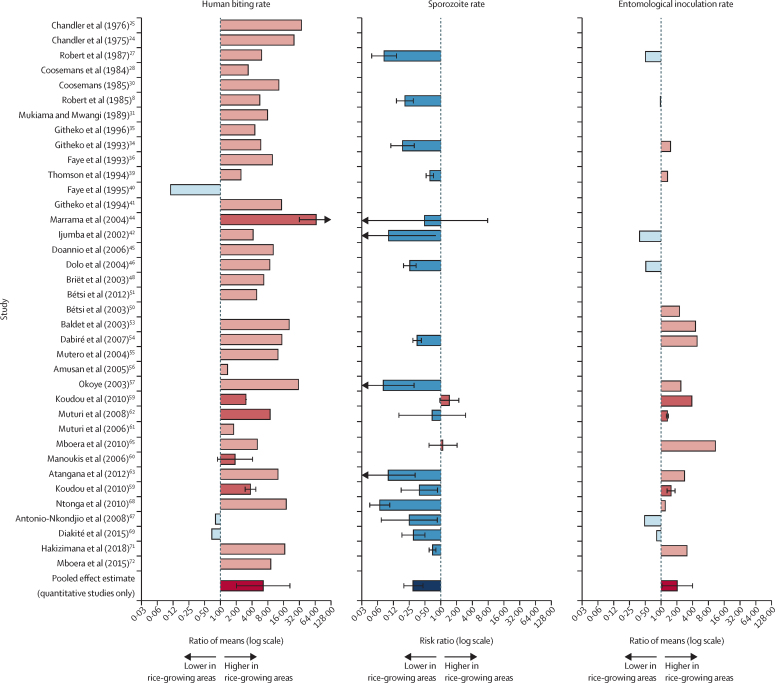
Meta-analyses of the association between residence in rice-growing areas and entomological outcomes Association of human biting rate, sporozoite rate, and entomological inoculation rate with rice-growing areas (as compared with non-rice-growing areas). Error bars are 95% CIs (for quantitative studies only). Studies are ordered by year of study. Semi-quantitative studies are represented by lighter-coloured bars and quantitative studies are represented by darker-coloured bars. Pooled effect estimates based on quantitative studies and calculated using random-effects models are presented at the bottom of each graph.

Meta-analysis of the four quantitative studies^44^, ^59^, ^60^, ^62^ that measured the human biting rate of *A gambiae* sl (from 1971 to 2016) showed a pooled effect (ROM) of 6·54 times (95% CI 1·99–21·46) higher human biting rate in rice-growing villages than in non-rice growing villages. Vector densities were consistently higher in rice-growing than in non-rice-growing communities (Figure 3, Figure 4). After taking into account 31 semiquantitative studies^8^, ^24^, ^25^, ^27^, ^28^, ^30^, ^31^, ^34^, ^35^, ^36^, ^39^, ^40^, ^41^, ^42^, ^45^, ^46^, ^48^, ^50^, ^53^, ^54^, ^55^, ^56^, ^57^, ^61^, ^63^, ^65^, ^67^, ^68^, ^69^, ^71^, ^72^ (those reporting crude effects without CIs), the median vector density in rice-growing villages was 34·0 bites per person per night (IQR 13·4–63·0), which is more than eight times greater than in non-rice villages (4·2 bites per person per night [1·0–12·8]). In the three most extreme cases, human biting rates were more than 30 times greater in rice-growing than in non-rice-growing villages.

*A gambiae* sl collected from rice-growing villages had 71% lower sporozoite rates than those found in non-rice-growing villages (RR 0·29 [95% CI 0·19–0·46], 17 studies).^8^, ^27^, ^34^, ^39^, ^42^, ^44^, ^46^, ^54^, ^57^, ^59^, ^62^, ^63^, ^65^, ^67^, ^68^, ^69^, ^71^

In quantitative studies that reported the entomological inoculation rate of *A gambiae* sl, this rate was doubled in rice-growing compared with non-rice-growing villages (ROM 2·03 [95% CI 1·02–4·06], two studies [three analyses]).^59^, ^62^ In semiquantitative studies, estimates of entomological inoculation rate were higher in rice-growing villages than in non-rice-growing villages in ten studies^34^, ^39^, ^50^, ^53^, ^54^, ^57^, ^63^, ^65^, ^68^, ^71^ and lower in six studies (figure 4).^8^, ^27^, ^42^, ^46^, ^67^, ^69^ Including the results from quantitative studies,^59^, ^62^ the proportion of studies in which the entomological inoculation rate was higher in rice-growing than in non-rice-growing villages was 68% (13 of 19 analyses, sign test p=0·1671).

Of the studies that reported the human biting rates of *A funestus* in rice-growing and non-rice-growing areas, only one was eligible as a quantitative study.^59^ In this study, an 89% lower abundance of *A funestus* was observed in rice villages (ROM 0·11 [95% CI 0·08–0·14]). A visualisation of the semiquantitative studies suggests a mixed effect: 13 studies^24^, ^25^, ^30^, ^31^, ^35^, ^44^, ^45^, ^50^, ^53^, ^57^, ^63^, ^65^, ^67^ found more *A funestus* in rice-growing areas than in non-rice areas whilst ten studies^8^, ^27^, ^28^, ^54^, ^56^, ^61^, ^62^, ^59^, ^69^, ^71^ found fewer *A funestus* (figure 3; appendix p 9).

Concerning shifts in the ratio of sibling species between rice-growing and non-rice-growing villages, only two studies (both conducted in Cameroon)^63^, ^67^ did not report a complete dominance of one species, and the constituent species did not change radically. We also looked for species shifts among *Anopheles* vectors and observed that, in west Africa,^8^, ^27^, ^36^, ^39^, ^40^, ^45^, ^46^, ^48^, ^50^, ^51^, ^53^, ^54^, ^56^, ^57^, ^59^, ^60^ the majority of vector populations in rice villages were *A gambiae* sl, while higher proportions of *A funestus* were found in control villages (figure 3). In east Africa, no conspicuous patterns were seen.^24^, ^25^, ^28^, ^30^, ^31^, ^34^, ^35^, ^41^, ^42^, ^65^, ^71^, ^72^

Risk of bias within individual cohort and cross-sectional studies was generally at an intermediate level (appendix pp 10–17). There was no evidence of publication bias in the meta-analysis of all outcomes except in malaria infection in the subgroup of studies done since 2003, where there was evidence of funnel plot asymmetry suggesting bias towards publication of positive findings (bias coefficient 2·82, p=0·0014; appendix pp 18–19). There were insufficient studies to test for asymmetry in the meta-analysis of entomological inoculation rate and clinical malaria. The GRADE approach indicated that the quality of evidence for the comparisons between rice-growing and non-rice-growing villages was very low (table).

**Table:** GRADE quality of evidence for the association between rice cultivation and epidemiological and entomological malaria outcomes

	**Summary of findings**		**Quality of evidence**					**Overall GRADE rating**
	Relative effect (95% CI)	Number of participants (studies)	Risk of bias	Inconsistency	Indirectness	Imprecision	Publication bias	
Human biting rate, *A gambiae* sl	ROM 6·54 (1·99–21·46)	823 (5)	Serious*: all studies were non-randomised and observational	Serious*: minimal overlap of confidence intervals and considerable heterogeneity (*I*^2^=99·97%, p<0·0001)	Serious: studies were conducted only in west and east Africa; these results might not be generalisable to central Africa	Serious*: at least one study showed a small number of events with wide 95% CIs	Not detected: Egger's test for bias found no evidence for funnel plot asymmetry (bias coefficient <1·00, p>0·05)	Very low: estimate is very uncertain
Sporozoite rate, *A gambiae* sl	RR 0·29 (0·19–0·46)	212 705 (18)	Serious*: all studies were non-randomised and observational	Serious*: minimal overlap of confidence intervals and considerable heterogeneity (*I*^2^=95·05%, p<0·0001)	Not serious: studies were conducted in a variety of sites in rural settings across sub-Saharan Africa; these findings are generalisable elsewhere	Serious*: at least one study showed a small number of events with wide 95% CIs	Not detected: Egger's test for bias found no evidence for funnel plot asymmetry (bias coefficient <1·00, p>0·05)	Very low: estimate is very uncertain
Entomological inoculation rate, *A gambiae* sl	ROM 2·03 (1·02–4·06)	2334 (3)	Serious*: all studies were non-randomised and observational	Serious*: minimal overlap of confidence intervals and considerable heterogeneity (*I*^2^=99·71%, p<0·0001)	Serious: studies were conducted only in west and east Africa; these results might not be generalisable to central Africa	Serious*: at least one study showed a small number of events with wide 95% CIs and there is uncertainty about the magnitude of effect of the intervention as it fails to exclude benefit or harm	Not detected: insufficient studies to construct funnel plots	Very low: estimate is very uncertain
Malaria infection, before 2003	RR 0·82 (0·63–1·06)	99 574 (16)	Serious*: all studies were non-randomised and observational	Serious*: minimal overlap of confidence intervals and considerable heterogeneity (*I*^2^=99·76%, p<0·0001)	Not serious: studies were conducted in a variety of sites in rural settings across sub-Saharan Africa; these findings are generalisable elsewhere	Serious*: at least one study showed a small number of events with wide 95% CIs and there is uncertainty about the magnitude of effect of the intervention as it fails to exclude benefit or harm	Not detected: Egger's test for bias found no evidence for funnel plot asymmetry (bias coefficient <1·00, p>0·05)	Very low: estimate is very uncertain
Malaria infection, after 2003	RR 1·73 (1·01–2·96)	14 002 (7)	Serious*: all studies were non-randomised and observational	Serious*: minimal overlap of confidence intervals and considerable heterogeneity (*I*^2^=99·18%, p<0·0001)	Serious: studies were conducted only in west and east Africa; these results might not be generalisable to central Africa	Serious*: there is uncertainty about the magnitude of effect of the intervention as it fails to exclude benefit or harm	Strongly suspected*: Egger's test for bias found some evidence for funnel plot asymmetry (bias coefficient 2·82, p=0·005)	Very low: estimate is very uncertain
Clinical malaria	IRR 0·71 (0·48–1·06)	77 890 (4)	Serious*: all studies were non-randomised and observational	Serious*: minimal overlap of confidence intervals and considerable heterogeneity (*I*^2^=92·73%, p<0·0001)	Serious: studies were conducted only in west and east Africa; these results might not be generalisable to central Africa	Serious*: there is uncertainty about the magnitude of effect of the intervention as it fails to exclude benefit or harm	Not detected: insufficient studies to construct funnel plots	Very low: estimate is very uncertain

Patient or population: people of all ages living in rural areas of malaria-endemic sub-Saharan Africa. Settings: Burkina Faso, Burundi, Cameroon, Côte d'Ivoire, Ghana, Kenya, Madagascar, Mali, Nigeria, Rwanda, Sierra Leone, Tanzania, The Gambia. Exposure: rice cultivation. GRADE=Grading of Recommendations, Assessment, Development and Evaluations. *A gambiae* sl=*Anopheles gambiae* sensu lato. ROM=ratio of means. RR=risk ratio. IRR=incidence rate ratio.

* Quality of evidence downgraded by 1 level.

## Discussion

To assess whether declining malaria rates in sub-Saharan Africa have changed the relationship between rice cultivation and malaria, we compared entomological and epidemiological malaria indicators between rice-growing and non-rice-growing villages using data from 53 observational studies. The results confirmed that before 2003, infection prevalence was not higher in rice-growing than in non-rice-growing communities. Conversely and most importantly, since 2003, prevalence was almost two times higher in rice-growing than in non-rice-growing communities. Additionally, the intensity of malaria transmission, measured as the entomological inoculation rate, tended to be higher in rice-growing areas: the lower sporozoite rates found in rice-dwelling *A gambiae* sl did not generally compensate for their greater numbers.

Previous reviews^6^, ^7^ based on studies done before 2003 showed that malaria prevalence was not higher in rice-growing than in non-rice-growing communities. Our re-examination of pre-2003 studies produced findings consistent with those reviews, and also showed that many sub-Saharan African countries had high malaria transmission intensities during this period. However, in more recent studies (applicable to the current malaria situation), we found higher infection prevalence in rice-growing than in non-rice-growing areas.

The differences between time periods could be explained by the introduction of the Roll Back Malaria initiative and the background developmental processes (general economic development, including housing) in Africa, both of which have changed the malaria picture in Africa drastically.^15^ In the past, rice-growing communities, compared with their non-rice-growing counterparts, tended to be wealthier and therefore had better socioeconomic conditions and access to drugs and mosquito nets, which might have constituted a protective factor against malaria.^76^ However, Roll Back Malaria brought about a massive upscaling of coverage of modern antimalaria interventions, including vector control, diagnostics, and treatment. Coverage has since become much more equitable within and between communities.^16^ Similarly, general development across the continent brought about better infrastructure, transport, and housing, as well as better health systems.^77^ Consequently, it can no longer be assumed that rice-growing villages have much better defences against malaria, or that non-rice-growing villages have no defences against the disease. It is presumed that the magnitude of change depends on which village characteristics were previously giving the differential protection between rice-growing and non-rice-growing villages; whether increased equity in antimalarial interventions or general development provided greater protection is a question that arises. As a consequence of the Roll Back Malaria initiative, there has also been a concomitant and equally widespread decline in the general intensity of transmission.^15^ Thus, the fraction of the population at risk who are exposed to high intensity transmission has substantially decreased. Many of those who were previously intensely exposed are now exposed only to low levels of transmission. Hence, the true differences in exposure between rice-growing and non-rice-growing villages are now observable in human clinical outcomes.

Overall, malaria vector densities were six times higher in rice-growing than non-rice-growing areas. This finding was expected, because the ecological conditions of the early stages of rice fields are exactly those preferred by larvae of *A gambiae* sl (fresh sunlit water of 2–10 cm depth, still or very slow-flowing, with silt or clay, without suspended organic matter, and non-deoxygenated).^3^, ^78^ However, the magnitude of difference is perhaps surprisingly high. The tendency for sporozoite rates to be lower in rice-growing areas is presumably due to density-dependent reductions in the vectorial capacity of the vector population,^9^ which could happen through a reduction in adult lifespan (eg, because of competition for food in the larval stage) or a reduction in adult feeding success (eg, because extreme biting nuisance drives most people to use bed nets).^10^, ^11^, ^12^, ^13^ There are also cases in which specific mechanisms dependent on unusual local conditions were operating. For instance, in Tanzania, there was evidence that the introduction of rice had removed the marshy breeding sites of *A funestus* (a very efficient vector) and replaced them with rice fields, which *A arabiensis* (a less efficient, although still important, vector) is better suited for breeding in.^42^ In one study in The Gambia, there were two annual crops of rice and two corresponding peaks of mosquito abundance, but only one annual peak of malaria transmission, which was during the rainy season. Apparently, during the hot dry season, vectors were abundant but not transmitting the parasite, either because they were too short-lived or because it was so hot that the parasites were killed inside the vectors.^79^

Previous reviews of whether rice-growing communities have a greater malaria burden have suggested that in rice-growing villages: (1) vector abundance tends to be higher; (2) sporozoite rates tend to be lower; and (3) the lower sporozoite rates compensate for the increased vector abundance, and there is no systematic tendency for malaria transmission to be more intense in rice-growing villages.^6^, ^7^ Our findings are consistent with (1) and (2), but not (3). Specifically, in 14 of the 19 studies, the reduction in sporozoite rate was not enough to compensate for the increase in vector abundance, and the pooled estimate suggests that malaria transmission in rice-growing villages tends to be about twice as intense as that in non-rice-growing villages. In other words, rice cultivation is, and apparently always was, associated with exposure to more intense transmission for unprotected people. It was never correct to assume that the mosquitoes from rice fields were numerous but somehow harmless.

This study has several limitations. First, it was based on observational studies, which can be subject to selection and information bias as well as confounders. Exposure and control groups might have low comparability: rice-growing and non-rice-growing communities could have been intrinsically different in their characteristics, even before the introduction of rice cultivation schemes (ie, there could be prerequisites that affect both malaria risk and the suitability of a village for irrigated rice fields). Second, observational studies can be prone to confounding because factors such as socioeconomic status, housing conditions, and access to health care (eg, antimalarial drugs and bed nets) are not always accounted for. Although we attempted to reduce confounding of this nature by presenting adjusted effect measures, very few studies reported them. The rating of very low quality of evidence according to the GRADE system indicates low confidence in the effect estimate.^23^ Nonetheless, we were not expecting, nor looking for, a true effect of rice cultivation on malaria risk; rather, we were more concerned about the direction of effect, which, although different in magnitude, was relatively consistent across studies given our a priori subgrouping. Third, a number of factors were not, and could not be, considered. Because of limited reporting, seasonality (wet *vs* dry, and seasonal *vs* perennial), intrinsic differences in landscapes of study sites, and characteristics of rice cultivation (type of rice grown, size of irrigation schemes, and distance of rice-growing communities from their fields) could not be accounted for. Control groups were also variable, each associated with different degrees of vector density. Additionally, of the seven post-2003 studies from which the pooled RR of malaria prevalence was calculated, three were done in central Tanzania by the same research group and could therefore be subject to bias.^64^, ^66^, ^73^

Considering that this review was based only on observational studies, it has highlighted the need for replicated studies comparing before and after the introduction of rice crops, and if possible, intervention studies to measure the effect of rice cultivation on malaria risk. Given the complex relationship between vector abundance, vectorial capacity, and malaria prevalence, future studies should include all entomological and epidemiological indicators to provide a clearer picture of the rice and malaria story. Such studies should also address questions of equity by including information on bed-net coverage, use of antimalarial drugs, socioeconomic factors, and housing.

Despite low-quality evidence, subgroup analyses comparing studies before and after the scale-up of malaria interventions suggested that this turning point has changed the rice–malaria relationship in Africa. Rice fields tend to produce large quantities of mosquitoes and, in most cases, any reduced vectorial capacity is inadequate to compensate for this increase in abundance, such that, on balance, there is greater exposure to infective mosquitoes in rice-farming communities. Thus, if we want to greatly expand rice cultivation in Africa and at the same time work towards malaria elimination, then we will need to develop ways to reconcile these two goals. In short, we need to find ways of growing rice without producing mosquitoes. Although various methods of controlling mosquitoes in rice fields have been studied, in most cases, these methods are only partially effective or are effective for only part of the season or in specific circumstances. What we need to know is how to combine these methods to provide effective control for the entire season and in wide variety of rice-growing settings.

## Data sharing

This manuscript makes use of publicly available data from published studies; therefore, no original data are available for sharing.

## Declaration of interests

We declare no competing interests.
