# Supplementary material for: Malaria transmission and prevalence in rice-growing versus non-rice-growing villages in Africa: a systematic review and meta-analysis
Source: Lancet Planet Health. 2022 Mar 9;6(3):e257–69. doi: 10.1016/S2542-5196(21)00349-1 (PMC8926871; doi:10.1016/S2542-5196(21)00349-1)
Supplement: Supplementary appendix [file mmc1.pdf]

THE LANCET
Planetary Health

Supplementary appendix

This appendix formed part of the original submission and has been peer reviewed. We post it as supplied by the authors.

Supplement to: Chan K, Tusting LS, Bottomley C, Saito K, Djouaka R, Lines J. Malaria transmission and prevalence in rice-growing versus non-rice-growing villages in Africa: a systematic review and meta-analysis. *Lancet Planet Health* 2022; **6:** e257–69.

Appendix

Supplementary Table 1. Search strategy in multiple databases (last search date = 18 September 2020)

Search Set	MEDLINE	EMBASE	Global Health	Web of Science
1	Exp malaria/	Exp malaria/	Exp malaria/	TS = malari*
2	Exp anopheles/	Exp Anopheles/	Exp anopheles/	TS = anophel*
3	Exp plasmodium/	Exp plasmodium/	Exp plasmodium/	TS = disease vector$
4	Malaria.tw.	Malaria.tw.	Malaria.ab.	TS = mosquito*
5	Malari*.tw.	Malari*.tw.	Malari*.ab.	TS = plasmodium
6	Anophel*.tw.	Anophel*.tw.	Anophel*.ab.	1 or 2 or 3 or 4 or 5
7	Mosquito*.tw.	Mosquito*.tw.	Mosquito*.ab.	TS = rice
8	Entomolog*.tw.	Entomolog*.tw.	Entomolg*.ab.	TS = "rice field$"
9	Parasitemi*.tw.	Parasitemi*.tw.	Parasitemi*.ab.	TS = "ricefield$"
10	Parasitaemi*.tw.	Parasitaemi*.tw.	Parasitaemi*.ab.	TS = "rice cultivat*"
11	Plasmodium.tw.	Plasmodium.tw.	Plasmodium.ab.	TS = "rice grow*"
12	1 or 2 or 3	1 or 2 or 3	1 or 2 or 3	TS = "rice padd*"
13	4 or 5 or 6 or 7 or 8 or 9 or 10 or 11	4 or 5 or 6 or 7 or 8 or 9 or 10 or 11	4 or 5 or 6 or 7 or 8 or 9 or 10 or 11	TS = "rice irrigat*"
14	12 or 13	12 or 13	12 or 13	7 or 9 or 10 or 11 or 12 or 13
15	Exp oryza/	Exp rice/	Exp rice/	6 and 15
16	Exp agriculture/	Exp agriculture/	Exp oryza/	
17	Rice.tw.	Exp "irrigation (agriculture)"	Exp agriculture/	
18	Rice field$.tw.	Rice.tw.	Rice.ab.	
19	Ricefield$.tw.	Rice field$.tw.	Rice field$.ab.	
20	Rice cultivat*.tw.	Ricefield$.tw.	Ricefield$.ab.	
21	Rice grow*.tw.	Rice adj4 cultivat*.tw.	Rice adj4 cultivat*.ab.	
22	Rice padd*.tw.	Rice adj4 grow*.tw.	Rice adj4 grow*.tw.	
23	Irrigat*.tw.	Rice adj4 practice$.tw.	Rice adj4 practice$.ab.	
24	15 or 16	Rice adj4 technique$.tw.	Rice adj4 technique$.ab.	
25	17 or 18 or 19 or 20 or 21 or 22 or 23	Rice adj2 padd*.tw.	Rice adj2 padd*.ab.	
26	24 or 25	Rice adj2 irrigat*.tw.	Rice adj2 irrigat*.ab.	
27	14 or 26	15 or 16 or 17	15 or 16 or 17	
28		18 or 19 or 20 or 21 or 22 or 23 or 24 or 25 or 26	18 or 19 or 20 or 21 or 22 or 23 or 24 or 25 or 26	
29		27 or 28	27 or 28	
30		14 and 29	14 and 29	

Supplementary Table 2. Characteristics of observational studies included in the quantitative, semi-quantitative and qualitative* analyses (n=53).

Study	Country	Year	Setting (irrigation scheme)	Primary vectors	Transmission / LLIN coverage / IRS coverage	Study design	Study size	Age group	Recruitment of participants / Method of mosquito collection	Control group(s) (distance from rice)	Follow-up	Outcomes included				
												Entomological			Epidemiological	
												HBR[a]	SR[a]	EIR[a]	PR[a]	MI[a]
**Chandler et al. 1975**[1]	Kenya	1971	Rural (Ahero)	*An. gambiae* s.l.	-	Cohort	7-22 catches per (3) village	N/A	HLC (inside and outside)	Grassland country (10 km)	12 months	Yes*				
**Chandler et al. 1976**[2]												Yes*				
**Audibert et al. 1990**[3]	Cameroon	1979	Rural	-	-	Repeated cross-sectional	4611	2 – 9	Random selection of clusters	Non-rice growing	N/A				Yes	
**Carnevale & Robert 1987**[4]	Burkina Faso	1980	Rural (Vallée du Kou)	*An. gambiae* s.l.	-	Cohort	-	N/A	-	Savannah (10 km)	9 months	Yes*	Yes	Yes*		
						Cross-sectional	2322	2 – 9	-		N/A				Yes	
**Coosemans et al. 1984**[5]	Burundi	1981	Rural (Rusizi)	*An. gambiae* s.l.	-	Cross-sectional	4-5 trap-nights per (9) village	N/A	HLC (inside and outside)	Market gardening, other crops e.g. cotton, banana, maize, yams	N/A	Yes*				
							-	0 – 20	-		N/A				Yes*	
**Couprie et al. 1985**[6]	Cameroon	1981	Rural	*An. gambiae* s.l.	55% prevalence	Cross-sectional	924	2 – 9	-	Next to lake, no rice cultivation	N/A				Yes	
**Coosemans 1985**[7]	Burundi	1982	Rural (Rusizi)	*An. gambiae* s.l.	-	Cohort	8-19 houses per (2) village	N/A	HLC (inside)	Cotton (15 km)	12 months	Yes*				
						Cross-sectional	3692	0 – 5	-		N/A				Yes	
**Robert et al. 1985**[8]	Burkina Faso	1983	Rural (Vallée du Kou)	*An. gambiae* s.l.	-	Cohort	176 captures across 4 villages	N/A	HLC (inside)	Savannah (20 km)	12 months	Yes*	Yes	Yes*		
**Mukiama & Mwangi 1989**[9]	Kenya	1984	Rural (Mwea)	*An. gambiae* s.l. & *An. pharoensis*	-	Cohort	2 houses fortnightly per (4) village	N/A	Based on permission of owner / PSC, CDCLT and exit traps	Periphery of rice area (5 km)	12 months	Yes*				
**Josse et al. 1987**[10]	Cameroon	1985	Rural	*An. gambiae* s.l.	-	Cross-sectional	2375	2 – 9	Sampling random clusters	Non-rice growing area	N/A				Yes	

Study	Country	Year	Setting (irrigation scheme)	Primary vectors	Transmission / LLIN coverage / IRS coverage	Study design	Study size	Age group	Recruitment of participants / Method of mosquito collection	Control group(s) (distance from rice)	Follow-up	Outcomes included				
												Entomological			Epidemiological	
												HBR[a]	SR[a]	EIR[a]	PR[a]	MI[a]
**Boudin et al. 1992**[11]	Burkina Faso	1985	Rural (Vallée du Kou)	*An. gambiae* s.l.	-	Cross-sectional	2120	0 – 14	Voluntary participation	Savannah	N/A				Yes	
**Githeko et al. 1993**[12]	Kenya	1989	Rural (Ahero)	*An. arabiensis*	-	Cohort	3 houses weekly per (2) village	N/A	HLC (inside and outside)	Sugar belt (6 km)	13 months	Yes*	Yes	Yes*		
**Githeko et al. 1996**[13]	Kenya	1989	Rural (Ahero)	*An. gambiae* s.l.	-	Cohort	2-3 houses monthly per (2) village	N/A	PSC	Sugar belt (6 km)	13 months	Yes*				
**Faye et al. 1993a**[14]	Senegal	1990	Rural	*An. gambiae* s.l.	-	Cohort	2 houses monthly per (3) village	N/A	HLC (inside and outside)	Traditional agriculture (5 km)	17 months	Yes*				
**Faye et al. 1993b**[15]					-	Cross-sectional	1149	0 – 9	-		N/A				Yes	
**Gbakima 1994**[16]	Sierra Leone	1991	Rural	Not reported	-	Cross-sectional	1106	All ages	Voluntary participation	Undeveloped swamps (5 km)	N/A				Yes	
**Thomson et al. 1994**[17]	The Gambia	1991	Rural	*An. gambiae* s.l.	-	Cohort	1 house weekly per (16) village	N/A	PSC and exit traps	Non-rice growing	7 months	Yes*	Yes	Yes*		
						Cross-sectional	1465	1 – 4	Compounds randomly selected, up to 30 children recruited from each 10 villages		N/A				Yes	
**Faye et al. 1995**[18]	Senegal	1992	Rural	*An. gambiae* s.l.	-	Cohort	156-168 trap-nights	0 – 10	HLC (inside and outside)	Traditional agriculture (5 km)	26 months	Yes*	Yes*			
						Cross-sectional	985	0 – 10	-		N/A				Yes	
**Githeko et al. 1994**[19]	Kenya	1993	Rural (Ahero)	*An. gambiae* s.l.	-	Cohort	41-65 trap-nights	N/A	CDCLT	Sugar belt (6 km)	-	Yes*				
**Ijumba et al. 2002a**[20]	Tanzania	1994	Rural (Lower Moshi)	*An. gambiae* s.l.	-	Cohort	2 houses fortnightly per (3) village	N/A	CDCLT	Savannah (8 km) and sugarcane irrigation	12 months	Yes*	Yes	Yes*		

Study	Country	Year	Setting (irrigation scheme)	Primary vectors	Transmission / LLIN coverage / IRS coverage	Study design	Study size	Age group	Recruitment of participants / Method of mosquito collection	Control group(s) (distance from rice)	Follow-up	Outcomes included: Entomological HBR[a]	SR[a]	EIR[a]	Epidemiological PR[a]	MI[a]
**Ijumba et al. 2002b[21]**						Cross-sectional	2951	1 – 4	All children enrolled	(15 km)	N/A				Yes	Yes
**Marrama et al. 2004[22]**	Madagas-car	1994	Rural	*An. arabiensis & An. gambiae s.s.*	-	Cohort	8-16 captures monthly per (3) village	N/A	HLC (inside and outside), PSC, CDCLT	Natural sub-arid ecosystem	12 – 36 months	Yes	Yes	Yes*		
**Doannio et al. 2006[23]**	Côte d'Ivoire	1994	Rural	*An. gambiae* s.l.	-	Cohort	4 houses (10-22 captures per village)	N/A	Selection based on group of dwellings / HLC (inside and outside)	Humid wooded savannah	10 months	Yes*				
**Dolo et al. 2004[24]**	Mali	1995	Rural	*An. gambiae* s.l.	-	Cohort	2 houses per (6) village	N/A	HLC (inside and outside)	Savannah (10 - 15 km)	30 months	Yes*	Yes	Yes*		
**Sissoko et al. 2004[25]**						Cross-sectional	9134	0 – 14	All children of appropriate age interviewed						Yes	Yes*
**Briet et al. 2003[26]**	Côte d'Ivoire	1996	Rural	*An. gambiae* s.l.	-	Cohort	Every 6 weeks per (13) village	N/A	HLC (inside and outside)	Inland valley without rice cultivation	12 months	Yes*				
**Henry et al. 2003[27]**	Côte d'Ivoire	1997	Rural	*An. gambiae* s.l.	4% used mosquito nets	Repeated cross-sectional	36217	All ages	Random selection of compounds within randomly selected villages	Lowlands with dense vegetation	N/A				Yes	
						Cohort	42818				10 months					Yes
**Betsi et al. 2003[28]**	Côte d'Ivoire	1998	Rural	Study concerns *An. funestus*	-	Cohort	3 houses per (3) village	N/A	HLC (inside and outside)	Lowlands with dense vegetation	12 months	Yes* (AF only)	Yes (AF only)	Yes* (AF, AG)		
**Betsi et al. 2012[29]**	Côte d'Ivoire	1998	Rural	*An. gambiae* s.l.	-	Cohort	3 houses every 6 weeks per (6) village	N/A	HLC (inside and outside)	Lowlands with dense vegetation	13 months	Yes*				
**Assi et al. 2013[30]**	Côte d'Ivoire	1998	Rural	*An. gambiae* s.l.	-	Repeated cross-sectional	29330	All ages	Random selection of villages	Inland valley without rice cultivation	N/A				Yes	
						Cohort	33678	All ages			12 months					Yes

Study	Country	Year	Setting (irrigation scheme)	Primary vectors	Transmission / LLIN coverage / IRS coverage	Study design	Study size	Age group	Recruitment of participants / Method of mosquito collection	Control group(s) (distance from rice)	Follow-up	Outcomes included				
												Entomological			Epidemiological	
												HBR[a]	SR[a]	EIR[a]	PR[a]	MI[a]
**Baldet et al. 2003**[31]	Burkina Faso	1999	Rural (Vallee du Kou)	*An. gambiae* s.l.	-	Cohort	4 houses monthly per (3) village	N/A	HLC (inside)	Savannah	12 months	Yes*		Yes*		
**Dabire et al. 2007**[32]	Burkina Faso	2000	Rural	Study concerns *An. funestus*	-	Cohort	4 houses weekly per (3) village	N/A	HLC (inside and outside)	Savannah (50 km)	5 months	Yes*	Yes	Yes*		
**Mutero et al. 2004**[33]	Kenya	2001	Rural (Mwea)	*An. arabiensis*	-	Cohort	12 houses monthly per (4) village	N/A	HLC (inside and outside)	Non-irrigated (16 km)	12 months	Yes*				
						Cross-sectional	206	0 – 9	All households with children <10 years of age identified and proportionately sampled		N/A				Yes	
**Amusan et al. 2005**[34]	Nigeria	2001	Rural	*An. gambiae* s.l.	-	Cohort	4 houses weekly per (2) village	N/A	CDCLT	Rubber & oil plantation within lowland forest	12 months	Yes*				
**Okoye 2003**[35]	Ghana	2002	Rural	*An. gambiae* s.l.	-	Cohort	4 houses monthly per (2) village	N/A	HLC (inside and outside) and PSC	Non-irrigated (10 km)	6 months	Yes*	Yes	Yes*		
**Koudou et al. 2009**[36]	Côte d'Ivoire	2002	Rural	*An. gambiae* s.l.	12% slept under a bednet	Repeated cross-sectional	3212	0 – 15	All children randomly selected from primary schools	Subsistence agriculture / intensive vegetable farming	36 months years				Yes	
**Koudou et al. 2010**[37]						Cohort	4 houses every 2 months per (2) village	N/A	HLC (inside and outside)			Yes	Yes	Yes		
**Manoukis et al. 2006**[38]	Mali	2004	Rural	*An. gambiae* s.l.	-	Cross-sectional	2 houses per (3) village	N/A	HLC (inside and outside)	Non-irrigated area (10 km)	N/A	Yes				
**Muturi et al. 2006**[39]	Kenya	2004	Rural (Mwea)	*An. arabiensis*	-	Cohort	30 houses fortnightly	N/A	Equal numbers of houses were selected from centre and periphery / HLC (inside and outside)	Other crops e.g. maize, beans, bananas (15 km)	12 months	Yes*				
**Muturi et al. 2008**[40]												Yes	Yes	Yes		

Study	Country	Year	Setting (irrigation scheme)	Primary vectors	Transmission / LLIN coverage / IRS coverage	Study design	Study size	Age group	Recruitment of participants / Method of mosquito collection	Control group(s) (distance from rice)	Follow-up	Outcomes included				
												Entomological			Epidemiological	
												HBR[a]	SR[a]	EIR[a]	PR[a]	MI[a]
**Atangana et al. 2012**[41]	Cameroon	2004	Rural	*An. arabiensis* & *An. gambiae* s.s.	-	Cohort	40 trap-nights per village	N/A	HLC (inside and outside) and PSC	Market gardening (200 km)	24 months	Yes*	Yes	Yes*		
**Rumisha et al. 2019**[42]	Tanzania	2004	Rural	*An. gambiae* s.l.	50%[b] / 75 – 85% slept under a mosquito net	Cross-sectional	7888	6 – 15	Primary schools within selected villages	Sugar (5 km) and savannah (15 km)	N/A				Yes	
**Mboera et al. 2010**[43]	Tanzania	2004	Rural	*An. gambiae* s.l.	-	Cohort	3 houses monthly per (5) village	N/A	House selection based on settlement patterns (and similar construction) / CDCLT	Sugar (5 km) and savannah (15 km)	12 months	Yes*	Yes	Yes*		
**Mboera et al. 2011**[44]	Tanzania	2005	Rural	*An. gambiae* s.l.	-	Cross-sectional	578	0 – 15	Schoolchildren (lower classes 1-4) from 6 primary schools	Sugar (5 km) and savannah (15 km)	N/A				Yes	
**Antonio-Nkondjio et al. 2008**[45]	Cameroon	2006	Rural	*An. arabiensis*, *An. gambiae* s.s. & *An. funestus*	-	Cohort	20-30 houses fortnightly per (3) village	N/A	HLC (inside and outside) and PSC	Other crops e.g. maize, millet, groundnut (20 km)	5 months	Yes*	Yes	Yes*		
**Ntonga et al. 2010**[46]	Cameroon	2006	Rural	*An. gambiae* s.l.	-	Cohort	3 houses monthly per (2) village	N/A	HLC (inside)	Rich in fish species	12 months	Yes*	Yes	Yes*		
**Diakite et al. 2015**[47]	Côte d'Ivoire	2007	Rural	*An. gambiae* s.l.	-	Repeated cross-sectional	4 sites monthly per (5) village	N/A	HLC (inside and outside)	Non-irrigated / not developed rice cultivation yet	33 months	Yes*	Yes	Yes*		
**Toure et al. 2016**[48]	Mali	2010	Rural	*An. gambiae* s.l.	40% [b] / 82% children below 10 slept under LLIN night prior survey	Cross-sectional	1145	0.5 – 9	Random selection of households. All children aged 6 months to 9 years enrolled to cohort study	Dry area where ground water pools depend on rainfall	N/A				Yes	
						Cohort	549				12 months					Yes

Study	Country	Year	Setting (irrigation scheme)	Primary vectors	Transmission / LLIN coverage / IRS coverage	Study design	Study size	Age group	Recruitment of participants / Method of mosquito collection	Control group(s) (distance from rice)	Follow -up	Outcomes included				
												Entomological			Epidemiological	
												HBR[a]	SR[a]	EIR[a]	PR[a]	MI[a]
**Hakizimana et al. 2018**[49]	Rwanda	2010	Rural	*An. gambiae* s.l.	-	Cohort	3 houses monthly per (21) village	N/A	HLC (inside and outside)	No rice cultivation	24 months	Yes*	Yes	Yes*		
**Mboera et al. 2015a**[50]	Tanzania	2012	Rural	*An. gambiae* s.l.	Over 83% of households had ITN	Cross-sectional	3 houses per (5) village	N/A	CDCLT	Dry / wet savannah (5-10 km)	N/A	Yes*	Yes*			
**Mboera et al. 2015b**[51]							1019	0 – 15	Schoolchildren were recruited		N/A				Yes	
**Hien et al. 2017**[52]	Burkina Faso	2014	Rural (Vallée du Kou)	*An. gambiae* s.l.	15-30%	Cross-sectional	614	0 – 15	Random sampling on individuals	Subsistence agriculture (15 km)	N/A				Yes	
**Babamale et al. 2020**[53]	Nigeria	2016	Rural	-	-	Cross-sectional	230	All ages	Voluntary participation based on study criteria	Sugar and yam	N/A				Yes	
											**Total (quantitative) =**	**4**	**17**	**2**	**22**	**4**
											**Total (semi-quantitative) =**	**31**		**16**		
											**Total (qualitative) =**		**2**	**1**	**1**	**1**
											**Total (qualitative, semi-quantitative & quantitative) =**	**36**	**19**	**19**	**23**	**5**
											**Total =**	**36**			**23**	
											**Total =**	**53**				

- = not reported
* = analysed qualitative / semi-quantitative
[a] HBR = human biting rate; SR = sporozoite rate; EIR = entomological inoculation rate; PR = parasite rate; MI = malaria incidence
[b] Prevalence considered for sample size estimation
HLC = human landing catch
CDCLT = CDC light trap
PSC = Pyrethrum spray catch

Supplementary Table 3. Meta-analyses of the association between residence in rice-growing areas and parasite prevalence (adjusted risk ratios).

Study	Country	Year	Rice type (cropping seasons)	Adjusted risk ratio (95% CI)
Henry et al. (2003)	Côte d'Ivoire	1997	Single	0.76 (0.66, 0.87)
Henry et al. (2003)	Côte d'Ivoire	1997	Double	0.54 (0.47, 0.62)
Assi et al. (2013)	Côte d'Ivoire	1998	Single	0.79 (0.64, 0.97)
Assi et al. (2013)	Côte d'Ivoire	1998	Double	0.85 (0.73, 1.00)
**Pooled effect estimate before 2003** ($p<0.0001$, $I^2=87.08\%$)				**0.73 (0.57, 0.89)**
Mboera et al. (2015)	Tanzania	2012	Single	7.69 (4.35, 14.29)
**Pooled effect estimate after 2003** ($p=1.0000$, $I^2=0.00\%$)				**7.69 (4.35, 14.29)**

Supplementary Table 4. Sensitivity analysis on the year 2003 as a cut-off point.

Year	Risk ratio pre-scale-up	Number of studies pre-scale-up	Risk ratio post-scale-up	Number of studies post-scale-up	Wald-type test p-value
2001	0.81 (0.61 – 1.08)	17	1.61 (0.99 – 2.60)	8	0.016
2002	0.82 (0.63 – 1.06)	18	1.73 (1.01 – 2.96)	7	0.014
2003	0.82 (0.63 – 1.06)	18	1.73 (1.01 – 2.96)	7	0.014
2004	0.87 (0.66 – 1.16)	19	1.62 (0.87 – 3.01)	6	0.075
2005	0.93 (0.71 – 1.22)	21	1.66 (0.64 – 4.29)	4	0.247

Supplementary Table 5. Meta-analyses of the association between residence in rice-growing areas and *An. gambiae* s.l. human biting rate.

Study	Country	Year	Control areas	Rice-growing areas	Ratio of means (95% CI)
Marrama et al. (2004)	Madagascar	1994	68.00	4534.60	66.69 (31.46, 141.37)
Koudou et al. (2010)	Côte d'Ivoire	2002	16.10	49.30	3.06 (2.97, 3,15)
Manoukis et al. (2006)	Mali	2004	23.00	43.67	1.90 (0.86, 4.17)
Muturi et al. (2008)	Kenya	2004	0.91	8.06	8.86 (8.75, 8.97)
Koudou et al. (2010)	Côte d'Ivoire	2005	10.30	38.45	3.73 (2.91, 4.79)
**Pooled effect estimate** ($p<0.0001$, $I^2=99.97\%$)					**6.54 (1.99, 21.46)**

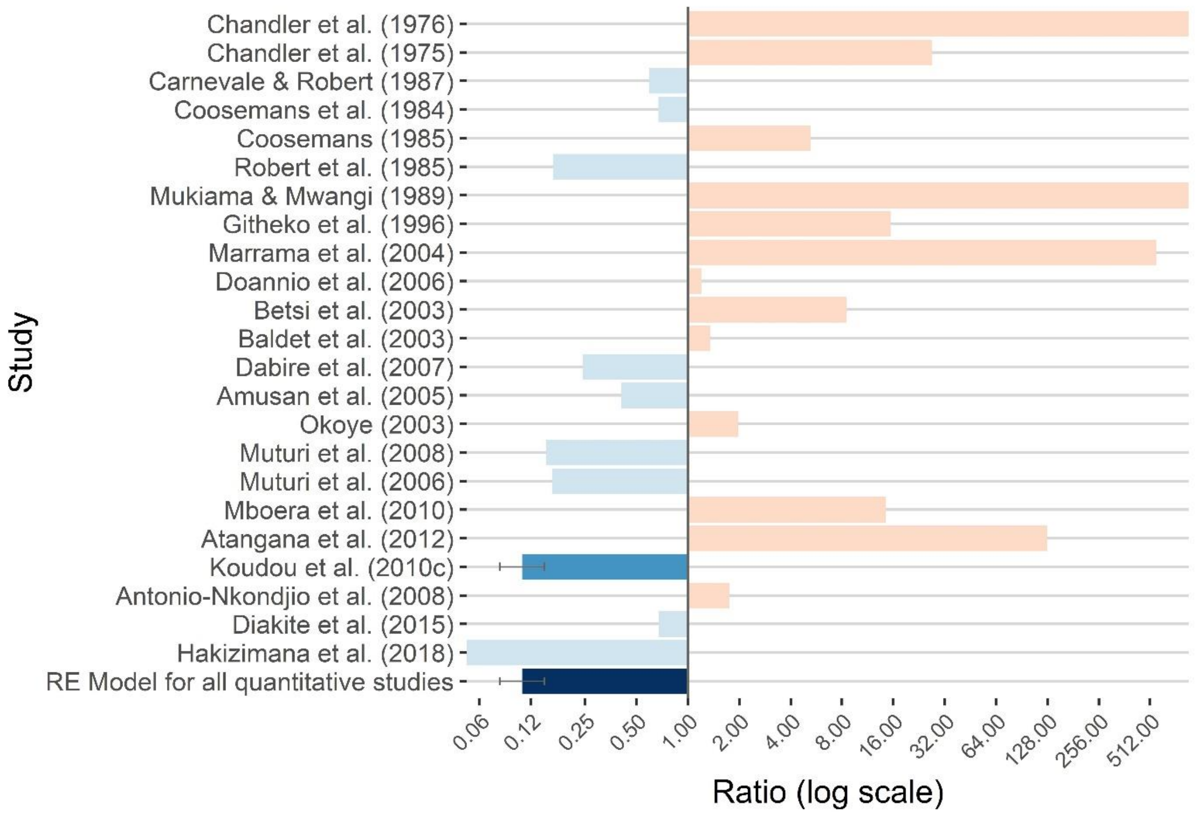

Supplementary Figure 2. Meta-analysis of the association between rice cultivation and *An. funestus* human biting rate. Ratio of human biting rate means (in rice areas compared to non-rice areas) and their 95% confidence intervals (only in quantitative studies, n=1, presented as error bars) are plotted according to year of study. Whilst light-coloured bars indicate semi-quantitative studies, solid-coloured bars indicate quantitative studies. Pooled effect estimates of quantitative studies are presented as dark-coloured bars at the bottom.

Supplementary Table 6A. Risk of bias assessment for studies with human biting rate included in the quantitative analysis (cohort studies, n=4).

Study		Selection				Comparability	Outcome			Overall quality assessment (max = 8)
		**Representativeness of the exposed group**	**Selection of the non-exposed group**	**Ascertainment of exposure (risk factor)**	**Demonstration that outcome of interest was not present at start of study**	**Comparability of groups on basis of the design or analysis**	**Assessment of outcome**	**Was follow-up long enough for outcomes to occur?**	**Adequacy of follow up of cohorts**	
	*	*a) Truly representative of the average individual or household in the community*	*a) Drawn from the same community as the exposed group*	*a) Validated measurement tool*	*a) Yes*	*a) Study controls for age*	*a) Validated measurement tool*	*a) Yes*	*a) Complete follow-up – all subjects accounted for*	
		b) Somewhat representative of the average individual or household in the community		*b) Structured interview*		*b) Study controls for socioeconomic status, bednet use or any additional factor*	*b) Record linkage*		*b) Subjects lost to follow-up unlikely to introduce bias*	
		c) Selected group of users e.g. nurses, volunteers	*b) Drawn from a different source*	*c) Written self-report*	*b) No*	*c) Study does not control for other factors*	*c) Self report*	*b) No*	*c) Follow up rate greater than 80% and no description of those lost*	
		d) No description of the derivation of the sample	*c) No description of the derivation of the non-exposed group*	*d) No description*			*d) No description*		*d) No statement*	
**Marrama et al. 2004**		b) *	b)	a) *	b)	c)	a) *	a) *	b) *	5
**Koudou et al. 2010**		b) *	b)	a) *	b)	c)	a) *	a) *	b) *	5
**Manoukis et al. (2006)**		b) *	b)	a) *	b)	c)	a) *	a) *	b) *	5
**Muturi et al. 2008**		b) *	b)	a) *	b)	c)	a) *	a) *	b) *	5

Supplementary Table 6B. Risk of bias assessment for studies with sporozoite rate included in the quantitative analysis (cohort studies, n=17).

Study		Selection				Comparability	Outcome			Overall quality assessment (max = 8)
		**Representativeness of the exposed group**	**Selection of the non-exposed group**	**Ascertainment of exposure (risk factor)**	**Demonstration that outcome of interest was not present at start of study**	**Comparability of groups on basis of the design or analysis**	**Assessment of outcome**	**Was follow-up long enough for outcomes to occur?**	**Adequacy of follow up of cohorts**	
	*	*a) Truly representative of the average individual or household in the community*	*a) Drawn from the same community as the exposed group*	*a) Validated measurement tool*	*a) Yes*	*a) Study controls for age*	*a) Validated measurement tool*	*a) Yes*	*a) Complete follow-up – all subjects accounted for*	
		b) Somewhat representative of the average individual or household in the community		*b) Structured interview*		*b) Study controls for socioeconomic status, bednet use or any additional factor*	*b) Record linkage*		*b) Subjects lost to follow-up unlikely to introduce bias*	
		c) Selected group of users e.g. nurses, volunteers	*b) Drawn from a different source*	*c) Written self-report*	*b) No*	*c) Study does not control for other factors*	*c) Self report*	*b) No*	*c) Follow up rate greater than 80% and no description of those lost*	
		d) No description of the derivation of the sample	*c) No description of the derivation of the non-exposed group*	*d) No description*			*d) No description*		*d) No statement*	
**Chandler et al. 1975**		b) *	b)	a) *	b)	c)	a) *	a) *	b) *	5
**Chandler et al. 1976**		b) *	b)	a) *	b)	c)	a) *	a) *	b) *	5
**Carnevale & Robert 1987**		b) *	b)	a) *	b)	c)	a) *	a) *	b) *	5
**Coosemans 1985**		b) *	b)	a) *	b)	c)	a) *	a) *	b) *	5
**Robert et al. 1985**		b) *	b)	a) *	b)	c)	a) *	a) *	b) *	5
**Mukiama & Mwangi 1989**		b) *	b)	a) *	b)	c)	a) *	a) *	b) *	5
**Githeko et al. 1993**		b) *	b)	a) *	b)	c)	a) *	a) *	b) *	5
**Githeko et al. 1996**		b) *	b)	a) *	b)	c)	a) *	a) *	b) *	5
**Faye et al. 1993a**		b) *	b)	a) *	b)	c)	a) *	a) *	b) *	5
**Thomson et al. 1994**		b) *	b)	a) *	b)	c)	a) *	a) *	b) *	5
**Faye et al. 1995**		b) *	b)	a) *	b)	c)	a) *	a) *	b) *	5
**Githeko et al. 1994**		b) *	b)	a) *	b)	c)	a) *	a) *	b) *	5
**Ijumba et al. 2002a**		b) *	b)	a) *	b)	c)	a) *	a) *	b) *	5
**Doannio et al. 2006**		b) *	b)	a) *	b)	c)	a) *	a) *	b) *	5
**Dolo et al. 2004**		b) *	b)	a) *	b)	c)	a) *	a) *	b) *	5
**Briet et al. 2003**		b) *	b)	a) *	b)	c)	a) *	a) *	b) *	5

Study		Selection				Comparability	Outcome			Overall quality assessment (max = 8)
		**Representativeness of the exposed group**	**Selection of the non-exposed group**	**Ascertainment of exposure (risk factor)**	**Demonstration that outcome of interest was not present at start of study**	**Comparability of groups on basis of the design or analysis**	**Assessment of outcome**	**Was follow-up long enough for outcomes to occur?**	**Adequacy of follow up of cohorts**	
	*	*a) Truly representative of the average individual or household in the community*	*a) Drawn from the same community as the exposed group*	*a) Validated measurement tool*	*a) Yes*	*a) Study controls for age*	*a) Validated measurement tool*	*a) Yes*	*a) Complete follow-up – all subjects accounted for*	
		b) Somewhat representative of the average individual or household in the community		*b) Structured interview*		*b) Study controls for socioeconomic status, bednet use or any additional factor*	*b) Record linkage*		*b) Subjects lost to follow-up unlikely to introduce bias*	
		c) Selected group of users e.g. nurses, volunteers	*b) Drawn from a different source*	*c) Written self-report*	*b) No*	*c) Study does not control for other factors*	*c) Self report*	*b) No*	*c) Follow up rate greater than 80% and no description of those lost*	
		d) No description of the derivation of the sample	*c) No description of the derivation of the non-exposed group*	*d) No description*			*d) No description*		*d) No statement*	
**Betsi et al. 2003**		b) *	b)	a) *	b)	c)	a) *	a) *	b) *	5
**Betsi et al. 2012**		b) *	b)	a) *	b)	c)	a) *	a) *	b) *	5
**Baldet et al. 2003**		b) *	b)	a) *	b)	c)	a) *	a) *	b) *	5
**Dabire et al. 2007**		b) *	b)	a) *	b)	c)	a) *	a) *	b) *	5
**Mutero et al. 2004**		b) *	b)	a) *	b)	c)	a) *	a) *	b) *	5
**Amusan et al. 2005**		b) *	b)	a) *	b)	c)	a) *	a) *	b) *	5
**Okoye 2003**		b) *	b)	a) *	b)	c)	a) *	a) *	b) *	5
**Koudou et al. 2010**		b) *	b)	a) *	b)	c)	a) *	a) *	b) *	5
**Muturi et al. 2008**		b) *	b)	a) *	b)	c)	a) *	a) *	b) *	5
**Muturi et al. 2006**		b) *	b)	a) *	b)	c)	a) *	a) *	b) *	5
**Mboera et al. 2010**		b) *	b)	a) *	b)	c)	a) *	a) *	b) *	5
**Manoukis et al. 2006**		b) *	b)	a) *	b)	c)	a) *	a) *	b) *	5
**Atangana et al. 2012**		b) *	b)	a) *	b)	c)	a) *	a) *	b) *	5
**Ntonga et al. 2010**		b) *	b)	a) *	b)	c)	a) *	a) *	b) *	5
**Antonio-Nkondjio et al. 2008**		b) *	b)	a) *	b)	c)	a) *	a) *	b) *	5
**Diakite et al. 2015**		b) *	b)	a) *	b)	c)	a) *	a) *	b) *	5
**Hakizimana et al. 2018**		b) *	b)	a) *	b)	c)	a) *	a) *	b) *	5

<table>
<tr><th rowspan="6">Study</th><th rowspan="2"></th><th colspan="4">Selection</th><th>Comparability</th><th colspan="3">Outcome</th><th rowspan="6">Overall quality assessment (max = 8)</th></tr>
<tr><th>Representativeness of the exposed group</th><th>Selection of the non-exposed group</th><th>Ascertainment of exposure (risk factor)</th><th>Demonstration that outcome of interest was not present at start of study</th><th>Comparability of groups on basis of the design or analysis</th><th>Assessment of outcome</th><th>Was follow-up long enough for outcomes to occur?</th><th>Adequacy of follow up of cohorts</th></tr>
<tr><td rowspan="2">*</td><td>a) Truly representative of the average individual or household in the community</td><td rowspan="2">a) Drawn from the same community as the exposed group</td><td>a) Validated measurement tool</td><td rowspan="2">a) Yes</td><td>a) Study controls for age</td><td>a) Validated measurement tool</td><td rowspan="2">a) Yes</td><td>a) Complete follow-up – all subjects accounted for</td></tr>
<tr><td>b) Somewhat representative of the average individual or household in the community</td><td>b) Structured interview</td><td>b) Study controls for socioeconomic status, bednet use or any additional factor</td><td>b) Record linkage</td><td>b) Subjects lost to follow-up unlikely to introduce bias</td></tr>
<tr><td rowspan="2"></td><td>c) Selected group of users e.g. nurses, volunteers</td><td>b) Drawn from a different source</td><td>c) Written self-report</td><td rowspan="2">b) No</td><td rowspan="2">c) Study does not control for other factors</td><td>c) Self report</td><td rowspan="2">b) No</td><td>c) Follow up rate greater than 80% and no description of those lost</td></tr>
<tr><td>d) No description of the derivation of the sample</td><td>c) No description of the derivation of the non-exposed group</td><td>d) No description</td><td>d) No description</td><td>d) No statement</td></tr>
<tr><td colspan="2">Mboera et al. 2015</td><td>b) *</td><td>b)</td><td>a) *</td><td>b)</td><td>c)</td><td>a) *</td><td>a) *</td><td>b) *</td><td>5</td></tr>
</table>

Supplementary Table 6C. Risk of bias assessment for studies with entomological inoculation rate included in the quantitative analysis (cohort studies, n=2).

Study		Selection				Comparability	Outcome			Overall quality assessment (max = 8)
		**Representativeness of the exposed group**	**Selection of the non-exposed group**	**Ascertainment of exposure (risk factor)**	**Demonstration that outcome of interest was not present at start of study**	**Comparability of groups on basis of the design or analysis**	**Assessment of outcome**	**Was follow-up long enough for outcomes to occur?**	**Adequacy of follow up of cohorts**	
	*	*a) Truly representative of the average individual or household in the community*	*a) Drawn from the same community as the exposed group*	*a) Validated measurement tool*	*a) Yes*	*a) Study controls for age*	*a) Validated measurement tool*	*a) Yes*	*a) Complete follow-up – all subjects accounted for*	
		b) Somewhat representative of the average individual or household in the community		*b) Structured interview*		*b) Study controls for socioeconomic status, bednet use or any additional factor*	*b) Record linkage*		*b) Subjects lost to follow-up unlikely to introduce bias*	
		c) Selected group of users e.g. nurses, volunteers	*b) Drawn from a different source*	*b) Written self-report*	*b) No*	*c) Study does not control for other factors*	*b) Self report*	*b) No*	*c) Follow up rate greater than 80% and no description of those lost*	
		d) No description of the derivation of the sample	*c) No description of the derivation of the non-exposed group*	*d) No description*			*d) No description*		*d) No statement*	
**Koudou et al. 2010**		b) *	b)	a) *	b)	c)	a) *	a) *	b) *	5
**Muturi et al. 2008**		b) *	b)	a) *	b)	c)	a) *	a) *	b) *	5

Supplementary Table 6D. Risk of bias assessment for studies with parasite prevalence included in the quantitative analysis (cross-sectional studies, n=22).

Study		Selection				Comparability	Outcome		Overall quality assessment (max = 9)
		**Representativeness of the sample**	**Sample size**	**Non-respondents**	**Ascertainment of exposure (risk factor)**	**Comparability of groups on basis of the design or analysis**	**Ascertainment of outcome**	**Statistical test**	
	**				*a) Validated measurement tool*		*a) Validated measurement tool*		
	*	*a) Truly representative of the average individual or household in the community*	*a) Justified and satisfactory (power calculation included)*	*a) Comparability between respondents and non-respondents characteristics is established, and the response rate is satisfactory*	*b) Non-validated measurement tool, but the tool is available or described*	*a) Study controls for age*	*b) Non-validated measurement method, but the method is available or described*	*a) The statistical test used to analyse the data is clearly described and appropriate, and the measurement of the association is presented, including confidence intervals and probability level*	
	*	*b) Somewhat representative of the average individual or household in the community*				*b) Study controls for socioeconomic status, bednet use or any additional factor*			
		c) Selected group of users e.g. nurses, volunteers	*b) Not justified*	*b) The response rate is unsatisfactory, or the comparability between respondents and non-respondents is unsatisfactory*	*c) No description of the measurement tool*	*c) Study does not control for other factors*	*c) No description of the measurement tool*	*b) The statistical test is not appropriate, not described or incomplete*	
		d) No description of the derivation of the sample		*c) No description of response rate or the characteristics or the responders and non-responders*					
**Audibert et al. 1990**		b) *	b)	c)	b) *	a) *	a) **	a) *	6
**Carnevale & Robert 1987**		b) *	b)	c)	b) *	c)	a) **	a) *	5
**Coosemans et al. 1984**		b) *	b)	c)	b) *	a) *	a) **	a) *	6
**Couprie et al. 1985**		b) *	b)	c)	b) *	c)	a) **	a) *	5
**Josse et al. 1987**		a) *	b)	c)	b) *	a) *	a) **	a) *	6
**Boudin et al. 1992**		b) *	b)	c)	b) *	a) *	a) **	a) *	6
**Faye et al. 1993b**		b) *	b)	c)	b) *	a) *	a) **	a) *	6
**Gbakima 1994**		b) *	b)	c)	b) *	c)	a) **	a) *	5
**Thomson et al. 1994**		b) *	b)	c)	b) *	c)	a) **	a) *	5
**Faye et al. 1995**		b) *	b)	c)	b) *	a) *	a) **	a) *	6
**Ijumba et al. 2002b**		b) *	b)	c)	a) **	a) *	a) **	a) *	7
**Sissoko et al. 2004**		b) *	b)	c)	b) *	a) *	a) **	a) *	6
**Henry et al. 2003**		a) *	a) *	c)	a) **	a) *	a) **	a) *	8
**Assi et al. 2013**		a) *	a) *	c)	a) **	a) *	a) **	a) *	8
**Mutero et al. 2004**		a) *	a) *	c)	b) *	a) *	a) **	a) *	7
**Koudou et al. 2009**		b) *	b)	c)	a) **	a) *	a) **	a) *	7
**Rumisha et al. 2019**		b) *	a) *	a) *	a) **	a) *	a) **	a) *	9
**Mboera et al. 2011**		c)	b)	c)	a) **	c)	a) **	a) *	5

**Study**		**Selection**				**Comparability**	**Outcome**		**Overall quality assessment (max = 9)**
		**Representativeness of the sample**	**Sample size**	**Non-respondents**	**Ascertainment of exposure (risk factor)**	**Comparability of groups on basis of the design or analysis**	**Ascertainment of outcome**	**Statistical test**	
	**				*a) Validated measurement tool*		*a) Validated measurement tool*		
	*	*a) Truly representative of the average individual or household in the community*<br>*b) Somewhat representative of the average individual or household in the community*	*a) Justified and satisfactory (power calculation included)*	*a) Comparability between respondents and non-respondents characteristics is established, and the response rate is satisfactory*	*b) Non-validated measurement tool, but the tool is available or described*	*a) Study controls for age*<br>*b) Study controls for socioeconomic status, bednet use or any additional factor*	*b) Non-validated measurement method, but the method is available or described*	*a) The statistical test used to analyse the data is clearly described and appropriate, and the measurement of the association is presented, including confidence intervals and probability level*	
		c) Selected group of users e.g. nurses, volunteers<br>*d) No description of the derivation of the sample*	*b) Not justified*	*b) The response rate is unsatisfactory, or the comparability between respondents and non-respondents is unsatisfactory*<br>*c) No description of response rate or the characteristics or the responders and non-responders*	*c) No description of the measurement tool*	*c) Study does not control for other factors*	*c) No description of the measurement tool*	*b) The statistical test is not appropriate, not described or incomplete*	
**Toure et al. 2016**		b) *	a) *	c)	a) **	a) and b) *	a) **	a) *	8
**Mboera et al. 2015b**		c)	b)	c)	a) **	a) *	a) **	a) *	6
**Hien et al. 2017**		b) *	a) *	c)	a) **	a) *	a) **	a) *	8
**Babamale et al. 2020**		b) *	b)	c)	b) *	c)	a) **	a) *	5

Supplementary Table 6E. Risk of bias assessment for studies with clinical malaria included in the quantitative analysis (cohort studies, n=4).

Study		Selection				Comparability	Outcome			Overall quality assessment (max = 8)
		**Representativeness of the exposed group**	**Selection of the non-exposed group**	**Ascertainment of exposure (risk factor)**	**Demonstration that outcome of interest was not present at start of study**	**Comparability of groups on basis of the design or analysis**	**Assessment of outcome**	**Was follow-up long enough for outcomes to occur?**	**Adequacy of follow up of cohorts**	
	*	*a) Truly representative of the average individual or household in the community*<br>*b) Somewhat representative of the average individual or household in the community*	*a) Drawn from the same community as the exposed group*	*a) Validated measurement tool*	*a) Yes*	*a) Study controls for age*<br>*b) Study controls for socioeconomic status, bednet use or any additional factor*	*a) Validated measurement tool*	*a) Yes*	*a) Complete follow-up – all subjects accounted for*<br>*b) Subjects lost to follow-up unlikely to introduce bias*	
		c) Selected group of users e.g. nurses, volunteers<br>*d) No description of the derivation of the sample*	*b) Drawn from a different source*<br>*c) No description of the derivation of the non-exposed group*	*b) Written self-report*<br>*d) No description*	*b) No*	*c) Study does not control for other factors*	*b) Self report*<br>*d) No description*	*b) No*	*c) Follow up rate greater than 80% and no description of those lost*<br>*d) No statement*	
**Ijumba et al. 2002b**		b) *	b)	a) *	b)	a) *	a) *	a) *	d)	5
**Henry et al. 2003**		a) *	b)	a) *	b)	a) *	a) *	a) *	d)	5
**Assi et al. 2013**		a) *	b)	a) *	b)	a) *	a) *	a) *	b) *	6
**Toure et al. 2016**		b) *	b)	a) *	b)	a) and b) *	a) *	a) *	b) *	6

A)

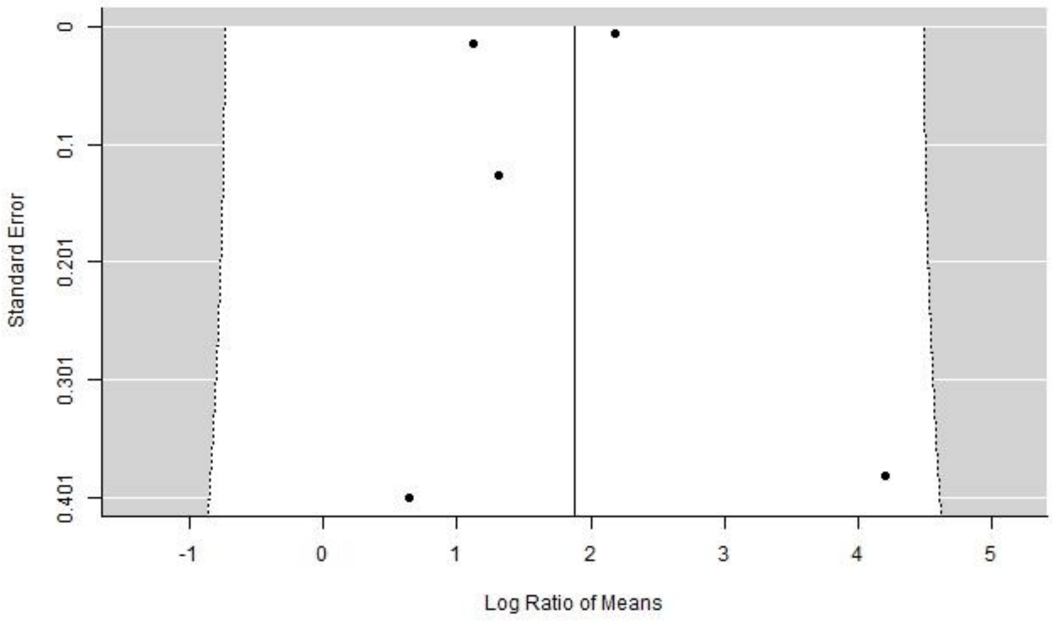

Standard Error
0
0.1
0.201
0.301
0.401
-1
0
1
2
3
4
5
Log Ratio of Means

B)

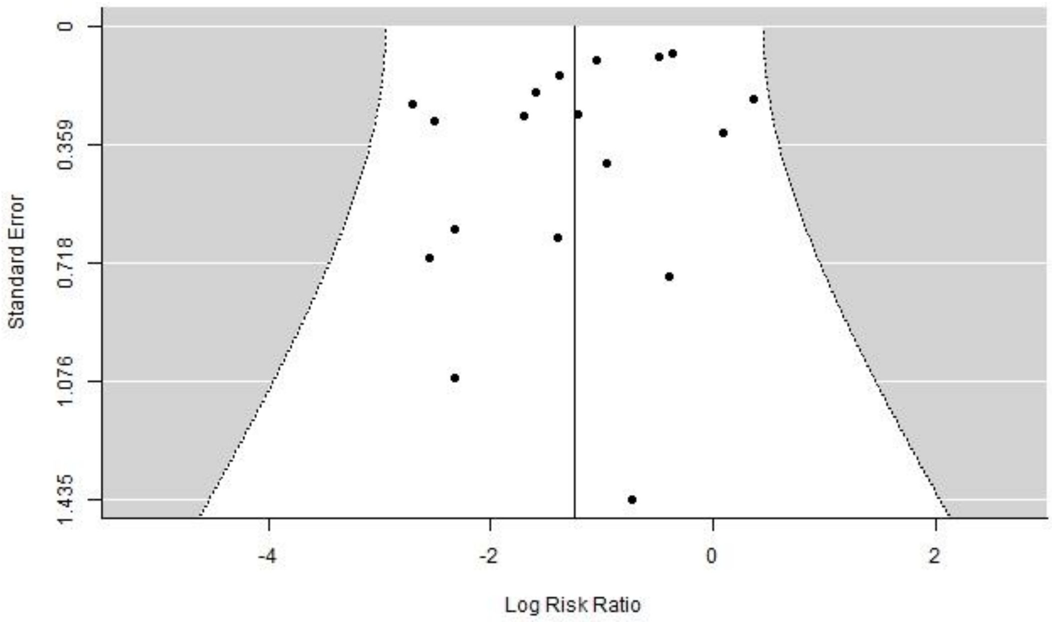

Standard Error
0
0.359
0.718
1.076
1.435
-4
-2
0
2
Log Risk Ratio

C)

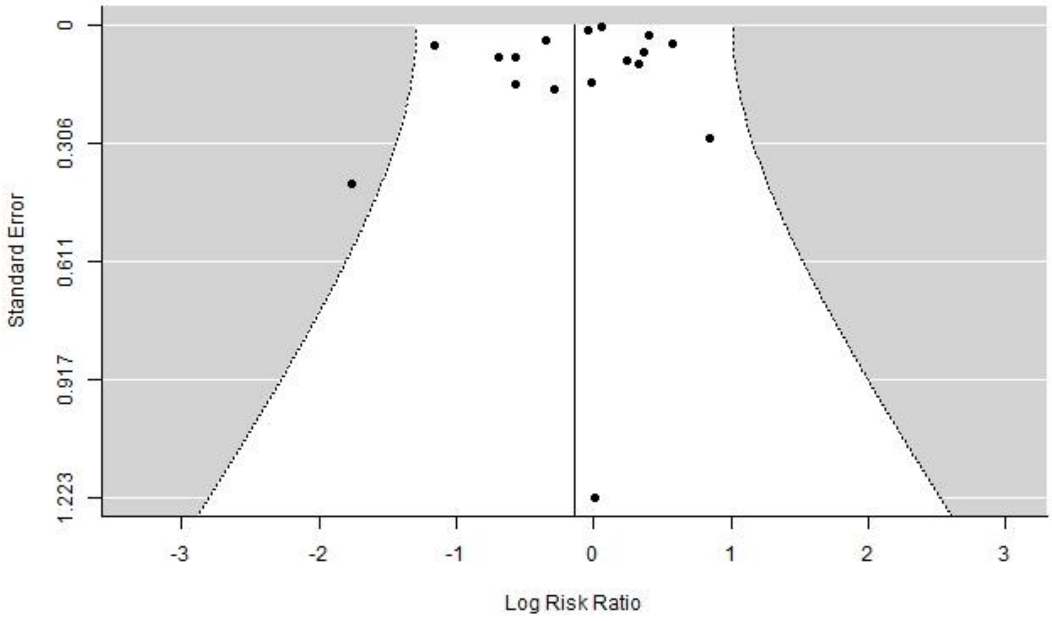

D)

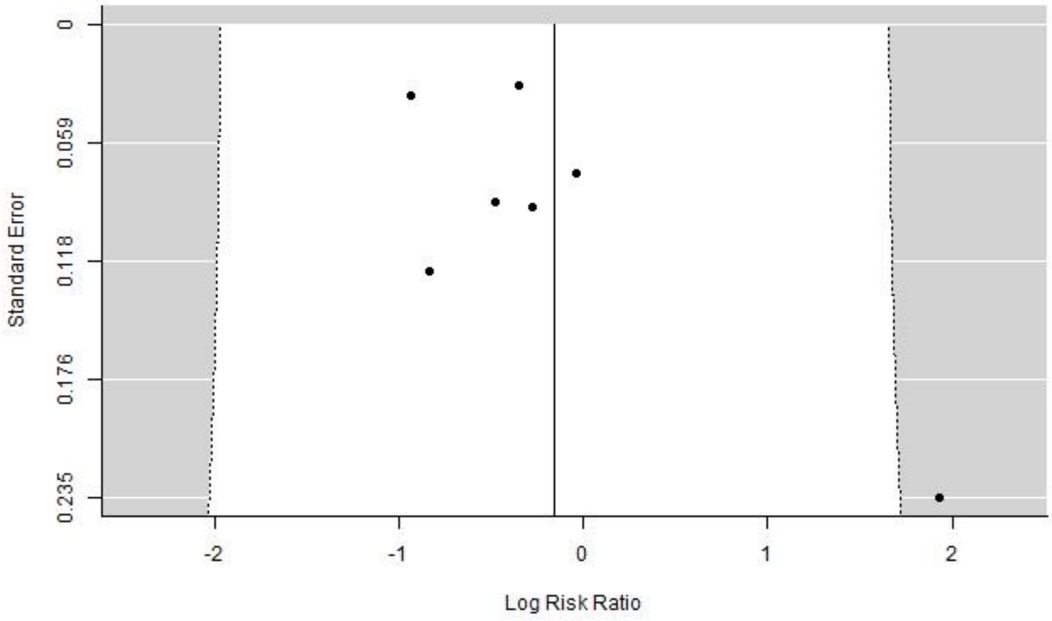

Supplementary Figure 3. Funnel plots assessing publication bias in the meta-analysis of malaria indicators in areas of rice vs. non-rice cultivation. The funnel plots illustrate the estimates of effect sizes against study size, and are used to detect publication bias. In the absence of publication bias, the plot creates a roughly funnel-shaped distribution. An asymmetric funnel indicates the possibility of publication bias, small study effects or selective outcome reporting. Plots show studies reporting (A) *An. gambiae* s.l. human biting rate (test for funnel plot asymmetry: z = 0.51, p = 0.61), (B) *An. gambiae* s.l. sporozoite rates (z = -0.90, p = 0.37), (C) parasite prevalence pre-2003 (z = -0.63, p = 0.53) and (D) parasite prevalence post-2003 (z = 3.19, p = 0.0014).

References

1 Chandler JA, Highton RB, Hill MN. Mosquitoes of the Kano Plain, Kenya. I. Results of indoor collections in irrigated and nonirrigated areas using human bait and light traps. *J Med Entomol* 1975; **12**: 504–10.

2 Chandler JA, Highton RB, Hill MN. Mosquitoes of the Kano Plain, Kenya. 2. Results of outdoor collections in irrigated and nonirrigated areas using human and animal bait and light traps. *J Med Entomol* 1976; **13**: 202–7.

3 Audibert M, Josseran R, Josse R, Adjidji A. Irragation, schistosomiasis, and malaria in the Logone Valley, Cameroon. *Am J Trop Med Hyg* 1990; **42**: 550–60.

4 Robert V, Carnevale P. Malaria transmission in three sites surrounding the area of Bobo-Dioulasso (Burkina Faso): the Savanna, a rice field, and the city. *Bull Soc Vector Ecol* 1987; **12**: 541–3.

5 Coosemans M, Wery M, Storme B, Hendrix L, Mfisi B. Malaria epidemiology in the Ruzizi plain, Burundi. *Epidemiol du Palud dans la plaine la Ruzizi, Burundi* 1984; **64**: 135–58.

6 Couprie B, Claudot Y, Same-Ekobo A. An epidemiological study of malaria in the rice-growing regions of Yagoua and Maga (north Cameroon). *Bull la Soc Pathol Exot* 1985; **78**: 191–204.

7 Coosemans MH. Comparaison de l'endémie malarienne dans une zone de riziculture et dans une zone de culture de coton dans la Plaine de la Rusizi, Burundi. *Ann Soc Belg Med Trop (1920)* 1985; **65 Suppl 2**: 187–200.

8 Robert V, Gazin P, Boudin C, Molez JF, Ouedraogo V, Carnevale P. The transmission of malaria in a wooded savanna zone and a rice-growing zone in the vicinity of Bobo Dioulasso (Burkina Faso). *La Transm du Palud en Zo savane arboree en Zo Rizic des environs Bobo Dioulasso (Burkina Faso)* 1985; **65**: 201–14.

9 Mukiama TK, Mwangi RW. Seasonal population changes and malaria transmission potential of Anopheles pharoensis and the minor anophelines in Mwea Irrigation Scheme, Kenya. *Acta Trop* 1989; **46**: 181–9.

10 Josse R, Josseran R, Audibert M, *et al.* Paludométrie et variations saisonnières du paludisme dans la région du projet rizicole de Maga (Nord Cameroun) et dans la région limitrophe. *Cah ORSTOM Série Entomol Médicale Parasitol* 1987; **numéro. sp**: 63–71.

11 Boudin C, Robert V, Carnevale P, Ambroise-Thomas P. Epidemiology of Plasmodium falciparum in a rice field and a savanna area in Burkina Faso. Comparative study on the acquired immunoprotection in native populations. *Acta Trop* 1992; **51**: 103–11.

12 Githeko AK, Service MW, Mbogo CM, Atieli FK, Juma FO. Plasmodium falciparum sporozoite and entomological inoculation rates at the Ahero rice irrigation scheme and the Miwani sugar-belt in western Kenya. *Ann Trop Med Parasitol* 1993; **87**: 379–91.

13 Githeko AK, Service MW, Mbogo CM, Atieli FK. Resting behaviour, ecology and genetics or malaria vectors in large scale agricultural areas of Western Kenya. *Parassitologia* 1996; **38**: 481–9.

14 Faye O, Fontenille D, Herve JP, Diack PA, Diallo S, Mouchet J. [Malaria in the Saharan region of Senegal. 1. Entomological transmission findings]. *Ann Soc Belg Med Trop (1920)* 1993; **73**: 21–30.

15 Faye O, Gaye O, Herve JP, Diack PA, Diallo S. Malaria in the Sahelian area of Senegal. 2. Parasitological indexes. *Ann Soc Belg Med Trop (1920)* 1993; **73**: 31–6.

16 Gbakima AA. Inland valley swamp rice development: Malaria, schistosomiasis, onchocerciasis in South Central Sierra Leone. *Public Health* 1994; **108**: 149–57.

17 Thomson MC, D'alessandro U, Bennett S, *et al.* Malaria prevalence is inversely related to vector density in The Gambia, West Africa. *Trans R Soc Trop Med Hyg* 1994; **88**: 638–43.

18 Faye O, Fontenille D, Gaye O, *et al.* Malaria and rice growing in the Senegal River delta. *Ann Soc Belg Med Trop (1920)* 1995; **75**: 179–89.

19 Githeko AK, Service MW, Mbogo CM, Atieli FK, Juma FO. Sampling Anopheles arabiensis, An gambiae sensu lato and An funestus (Diptera, Culicidae) with CDC light traps near a rice irrigation area and a sugarcane belt in Western Kenya. *Bull Entomol Res* 1994; **84**: 319–24.

20 Ijumba JN, Mosha FW, Lindsay SW. Malaria transmission risk variations derived from different agricultural practices in an irrigated area of northern Tanzania. *Med Vet Entomol* 2002; **16**: 28–38.

21 Ijumba JN, Shenton FC, Clarke SE, Mosha FW, Lindsay SW. Irrigated crop production is associated with less malaria than traditional agricultural practices in Tanzania. *Trans R Soc Trop Med Hyg* 2002; **96**: 476–80.

22 Marrama L, Jambou R, Rakotoarivony I, *et al.* Malaria transmission in Southern Madagascar: Influence of the environment and hydro-agricultural works in sub-arid and humid regions: Part 1. Entomological investigations. *Acta Trop* 2004; **89**: 193–203.

23 Doannio JM, Dossou-Yovo J, Diarrassouba S, Rakotondraibe ME, Chauvancy G, Riviere F. [Comparison of specific composition and mosquitoes population dynamics in two villages of the centre of Côte d'Ivoire, with and without irrigated rice growing area]. *Bull la Soc Pathol Exot* 2006; **99**: 204–6.

24 Dolo G, Briët OJT, Dao A, *et al.* Malaria transmission in relation to rice cultivation in the irrigated Sahel of Mali. *Acta Trop* 2004; **89**: 147–59.
25 Sissoko MS, Dicko A, Briët T, *et al.* Malaria incidence in relation to rice cultivation in the irrigated Sahel of Mali. *Acta Trop* 2004; **89**: 161–70.
26 Briët OJT, Dossou-Yovo J, Akodo E, Van De Giesen N, Teuscher TM. The relationship between Anopheles gambiae density and rice cultivation in the savannah zone and forest zone of Côte d'Ivoire. *Trop Med Int Heal* 2003; **8**: 439–48.
27 Henry MC, Rogier C, Nzeyimana I, *et al.* Inland valley rice production systems and malaria infection and disease in the savannah of Côte d'Ivoire. *Trop Med Int Heal* 2003; **8**: 449–58.
28 Betsi N, Koua H, FouaBi K. Anopheles funestus (Giles, 1900), la riziculture et la paludisme dans la region forestiere ouest de la Cote d'Ivoire. *Cah Agric* 2003; **12**: 341–6.
29 Betsi AN, Tchicaya ES, Koudou BG. High proliferation of An. gambiae and An. funestus larvae in irrigated and non-irrigated rice fields in the Western forest region of Côte d'Ivoire. *Bull la Soc Pathol Exot* 2012; **105**: 220–9.
30 Assi S-B, Henry M-C, Rogier C, *et al.* Inland valley rice production systems and malaria infection and disease in the forest region of western Côte d'Ivoire. *Malar J* 2013; **12**: 233.
31 Baldet T, Diabate A, Guiguemde TR. [Malaria transmission in 1999 in the rice field area of the Kou Valley (Bama), (Burkina Faso)]. *Sante* 2003; **13**: 55–60.
32 Dabiré KR, Baldet T, Diabaté A, *et al.* Anopheles funestus (Diptera: Culicidae) in a humid savannah area of western Burkina Faso: bionomics, insecticide resistance status, and role in malaria transmission. *J Med Entomol* 2007; **44**: 990–7.
33 Mutero CM, Kabutha C, Kimani V, *et al.* A transdisciplinary perspective on the links between malaria and agroecosystems in Kenya. *Acta Trop* 2004; **89**: 171–86.
34 Amusan AAS, Mafiana CF, Idowu AB, Olatunde GO. Sampling mosquitoes with CDC light trap in rice field and plantation communities in Ogun State, Nigeria. *Tanzan Health Res Bull* 2005; **7**: 111–6.
35 Okoye N. The Impact of an Irrigation Scheme on Malaria Transmission by Anopheles (Diptera: Culicidae) In A Coastal Savanna Area of Ghana. 2003.
36 Koudou BG, Tano Y, Keiser J, *et al.* Effect of agricultural activities on prevalence rates, and clinical and presumptive malaria episodes in central Côte d'Ivoire. *Acta Trop* 2009; **111**: 268–74.
37 Koudou BG, Doumbia M, Janmohamed N, *et al.* Effects of seasonality and irrigation on malaria transmission in two villages in Cote d'Ivoire. *Ann Trop Med Parasitol* 2010; **104**: 109–21.
38 Manoukis NC, Touré MB, Sissoko I, *et al.* Is vector body size the key to reduced malaria transmission in the irrigated region of Niono, Mali? *J Med Entomol* 2006; **43**: 820–7.
39 Muturi EJ, Shililu J, Jacob B, Gu W, Githure J, Novak R. Mosquito species diversity and abundance in relation to land use in a riceland agroecosystem in Mwea, Kenya. *J Vector Ecol* 2006; **31**: 129–37.
40 Muturi EJ, Muriu S, Shililu J, *et al.* Effect of rice cultivation on malaria transmission in central Kenya. *Am J Trop Med Hyg* 2008; **78**: 270–5.
41 Atangana J, Fomena A, Tamesse JL, Fondjo E. Pratiques agricoles et épidémiologie du paludisme en zone soudano-sahélienne du Cameroun. *Bull la Soc Pathol Exot* 2012; **105**: 23–9.
42 Rumisha SF, Shayo EH, Mboera LEG. Spatio-temporal prevalence of malaria and anaemia in relation to agro-ecosystems in Mvomero district, Tanzania. *Malar J* 2019; **18**: 1–14.
43 Mboera LEG, Senkoro KP, Mayala BK, *et al.* Spatio-temporal variation in malaria transmission intensity in five agro-ecosystems in Mvomero district, Tanzania. *Geospat Health* 2010; **4**: 167–78.
44 Mboera LEG, Senkoro KP, Rumisha SF, Mayala BK, Shayo EH, Mlozi MRS. Plasmodium falciparum and helminth coinfections among schoolchildren in relation to agro-ecosystems in Mvomero District, Tanzania. *Acta Trop* 2011; **120**: 95–102.
45 Antonio-Nkondjio C, Atangana J, Ndo C, *et al.* Malaria transmission and rice cultivation in Lagdo, northern Cameroon. *Trans R Soc Trop Med Hyg* 2008; **102**: 352–9.
46 Ntonga PA, Bakwo EFM, Kekeunou S, Belong P, Messi J. Impact of extensive rice cultivation on the culicid fauna and the transmission of malaria in Tonga, West Cameroon. *J Anim Plant Sci* 2010; **7**: 841–51.
47 Diakité NR, Guindo-Coulibaly N, Adja AM, *et al.* Spatial and temporal variation of malaria entomological parameters at the onset of a hydro-agricultural development in central Côte d'Ivoire. *Malar J* 2015; **14**: 340.
48 Touré M, Sanogo D, Dembele S, *et al.* Seasonality and shift in age-specific malaria prevalence and incidence in Binko and Carrière villages close to the lake in Selingué, Mali. *Malar J* 2016; **15**. DOI:10.1186/s12936-016-1251-4.
49 Hakizimana E, Karema C, Munyakanage D, *et al.* Spatio-temporal distribution of mosquitoes and risk of malaria infection in Rwanda. *Acta Trop* 2018; **182**: 149–57.
50 Mboera LEG, Bwana VM, Rumisha SF, Stanley G, Tungu PK, Malima RC. Spatial abundance and human

biting rate of Anopheles arabiensis and Anopheles funestus in savannah and rice agro-ecosystems of Central Tanzania. *Geospat Health* 2015; **10**: 322.

51 Mboera LEG, Bwana VM, Rumisha SF, *et al.* Malaria, anaemia and nutritional status among schoolchildren in relation to ecosystems, livelihoods and health systems in Kilosa District in central Tanzania. *BMC Public Health* 2015; **15**. DOI:10.1186/s12889-015-1932-x.

52 Hien AS, Sangaré I, Coulibaly S, *et al.* Parasitological indices of malaria transmission in children under fifteen years in two ecoepidemiological zones in southwestern Burkina Faso. *J Trop Med* 2017; **2017**. DOI:10.1155/2017/1507829.

53 Babamale OA, Opeyemi OA, Bukky AA, *et al.* Association between farming activities and plasmodium falciparum transmission in rural communities in Nigeria. *Malaysian J Med Sci* 2020; **27**: 105–16.
